# Formation of Volatile and Aroma Compounds during the Dehydration of Membrane-Clarified Sugarcane Juice to Non-Centrifugal Sugar

**DOI:** 10.3390/foods10071561

**Published:** 2021-07-05

**Authors:** Yanjing Ge, Kai Li, Caifeng Xie, Yongshi Xu, Changrong Shi, Fangxue Hang, William O. S. Doherty

**Affiliations:** 1 College of Light Industry and Food Engineering, Guangxi University, Nanning 530004, China; gyjpapers@163.com (Y.G.); gxlikai@gxu.edu.cn (K.L.); fcx11@163.com (C.X.); 2 Provincial and Ministerial Collaborative Innovation Center for Sugar Industry, Nanning 530004, China; 3 Engineering Research Center for Sugar Industry and Comprehensive Utilization, Ministry of Education, Nanning 530004, China; xuyongsih@163.com; 4 Guangxi Baiguitang Food Technology Co., Ltd., Guigang 537321, China; 5 Centre for Agriculture and the Bioeconomy, Faculty of Science, Queensland University of Technology, Brisbane, QLD 4000, Australia; w.doherty@qut.edu.au; 6 School of Mechanical, Medical and Process Engineering, Faculty of Engineering, Queensland University of Technology, Brisbane, QLD 4000, Australia

**Keywords:** NCS (non-centrifugal sugar), membrane-clarified sugarcane juice, volatiles development, chemometrics, volatiles precursors

## Abstract

The development of volatile compounds and their precursors during the dehydration process of membrane-clarified sugarcane juice to non-centrifugal sugar (NCS) was investigated. Head-space solid phase microextraction/gas chromatography–mass spectrometry (HS-SPME/GC–MS) coupled with chemometrics was employed to assess the differences at the various stages of the dehydration process. A total of 111 volatile compounds were identified, among which 57 were endogenous compounds from sugarcane juice and displayed an attenuated abundance in the first 30 min. Typical oxygen and nitrogen heterocyclic compounds, including furans and pyrazines, and aldehydes derived were found to be the main volatiles contributing to the formation of NCS characteristic aroma, with phenols, alcohols, esters, acids, and sulfur compounds as supplementary odor. Free amino acids and reducing sugars were identified as important precursors for the aroma development process. The low temperature (90–108 °C) and micro vacuum condition (−0.03 MPa) approach used in this study could be an alternative option for the manufacture of NCS.

## 1. Introduction

Non-centrifugal sugar (NCS) is a typical unrefined sugar produced by dehydrating sugarcane juice without centrifugation. As many of the micronutrients and bioactive components, such as phenolics, minerals, proteins, amino acids, and long chain lipids (policosanol, steroids, terpenoids etc.), are not removed during the manufacturing process of NCS, its nutritional and biological properties have been widely investigated [1,2,3]. Today, NCS is used as an alternative for table sugar since refined/purified sugar has been linked to several health problems like obesity, dental caries, and cardiovascular disease [4,5]. Apart from the nutritional and bio-functional attributes, the unique aroma properties of NCS have extended its scope of applications to confectionery, bakery, and beverage products [6,7,8].

In general, NCS is produced from clarified sugarcane juice, through evaporation to a water content to 88–94% (dehydration process), followed by crystallization and drying [1]. The presence of the aroma in NCS has been linked to two main sources, sugarcane plant and the volatile components developed during the manufacturing and storage processes [9,10]. The main sources of volatile compounds in the NCS manufacturing process are attributed to the thermal degradation of carbohydrates via caramelization reactions and/or the Maillard reaction. The Maillard reaction involves a complex reaction between amino acids and reducing sugars and has been identified as the major contributor to the aroma development process [11]. In addition to Maillard reaction products (MRPs), other volatiles, including acids, alcohols, phenolics, esters, sulfur compounds etc., also contribute to the overall aroma quality and characteristic.

To understand the formation of aroma profiles of NCS and determine formulations of NCS-based food and beverage products, many studies have been conducted to identify the aroma components in sugarcane juice [12,13,14], commercial NCS products [7,15,16,17], and NCS beverages at different brixes [8], and the impacts of the storage process on the NCS aroma properties [6].

Among the various steps in the NCS manufacturing process, the dehydration process is the key step where aroma products are developed, contributing significantly to the overall aroma quality of the final product. Currently, the dehydration process under atmospheric condition is adopted worldwide, including the open pan (OP), the horizonal thin film (HTF), and the vertical thin film (VTF) drying processes. Asikin and co-authors (2016) [10] systematically studied the impacts of the three drying-solidification processes on the physical and the antioxidant properties of NCS products and the fractions of the volatiles present in them. It was shown that NCS derived from HTF and VTF processes (132–135 °C) contains a greater proportion of aroma components than that from the OP (128–132 °C) process. This was attributed to the larger upper surface area of the OP process which enhanced the release of aroma components to the environment. The higher temperature in both HTF and VTF enhance the formation of the MRPs and the cylindrical shape of the evaporator tubes prevents aroma compounds from vaporization and allows entrapment of these compounds in the solidified sugar products.

In addition to temperature, the production of MRPs is also depend on pH, water activity, type of substrate species, and substrate concentration [6,18]. Clarification is a process that removes the non-sugars impurities, such as proteins and polysaccharides etc., that affect aroma formation in the subsequent concentration/dehydration process [9]. The membrane filtration process has been demonstrated as a superior clarification process to the traditional chemical processes for NCS production (M-NCS), as a higher proportion of nutritional and antioxidation components were found with the M-NCS [1]. A preliminary analysis of the volatile flavor components in M-NCS has been carried out [1]. This may not only be related to differences in the juice composition between the traditional and membrane clarification processes, but to the lower boiling temperature maintained under micro vacuum condition used in the membrane clarified juice, as opposed to the OP drying process used in the clarified juice derived by traditional means.

Studies on the aroma profiles of NCS have mainly focused on the final product [1,6,7,15,16,17]. To the best of our knowledge, we are not aware of any reports on the changes of the aroma profile during the dehydration process of membrane clarified cane juice at a relatively low temperature. In the present study, we have studied the dynamic changes of the proportion of volatile components that occurred during the dehydration process during the production of NCS derived from membrane clarified juice. The dehydration process was conducted at 90–108 °C with a vacuum of 225 Torr and up to 240 min to simulate the production process at a Chinese sugar factory. The atmospheric dehydration processes require higher energy input, while the micro vacuum dehydration process could on one hand utilize the existing multiple effect evaporator system on-site [19], and increase the energy efficiency through using vapor as the heating source for the remaining processes in the factory. The outputs from this study will allow future research to design new dehydration schemes in order to tune the aroma composition to a desired specification and ensure good quality control for NCS products.

## 2. Materials and Methods

### 2.1. Standards and Reagents

Sugar standards (sucrose, glucose, and fructose) and *n*-alkanes (C8–C40) were purchased from Sigma Corporation (Shanghai, China). (s)-(+)-2-Octanol was purchased from Alfa Aesar Corporation (Shanghai, China). Mixed amino acids standard solution was obtained from Wako Pure Chemical Industries, Limited. (Tokyo, Japan, 99.9%). Sodium chloride were purchased from Kelong Chemical Industry (Chengdu, China).

### 2.2. Simulated NCS Production and the Sampling Procedure

To simulate the membrane clarification based NCS production process in Bai Gui Tang Food Company, micro-vacuum boiling (225 Torr) process with the rotary vacuum evaporator were used for NCS preparation at laboratory scale. Sugarcane stems were manually harvested and squeezed with a laboratory three-roller crusher (Jiangmen Machinery Factory, Jiangmen, China). Milk of lime (slaking from CaO, which was purchased from Damao Chemical Reagent company, Tianjin, China) was used to bring up the juice pH to ~7.0 and heated to 90 °C. A 50-nm ceramic membrane was used to clarify the juice. The fresh clarified juice was designated as the 0 min sample, of which the detected volatile profiles represent the endogenous sugarcane juice flavor. The juice was boiled under micro vacuum condition, the real-time temperature of the samples were monitored with an internal induction thermometer (90 °C to108 °C), and aliquot samples were taken every 30 min for 240 min. Each sample was split into two aliquots, one of which was analyzed directly to define the volatile profiles. The other aliquot was stored at −20 °C for sugars and amino acids analysis.

### 2.3. Extraction and Analysis of Volatile Components

The volatile compounds of each processing sample were extracted using headspace-solid phase micro extraction (HS-SPME) adopted from Asikin et al. (2014) [6] with some modifications. A 50/30 μm fiber coated with DVB/CAR/PDMS (Supelco, St. Louis, MO, USA) was used as the hand-held SPME fiber, and was kept in the GC-MS injection port for 30 min at 250 °C prior to use to prevent the contamination. For the detailed extraction procedures please refer to the supplementary.

An Agilent gas chromatograph (GC 7890B, Agilent, Santa Clara, CA, USA) equipped with a 5977A mass spectrometer detector system was employed for volatile analysis according to the method from Asikin et al. (2014) [6] and Ma et al. (2018) [20] with some modifications. The aroma extracts were injected to DB-wax capillary column (60 m × 0.32 mm i.d., film thickness 0.25 μm, Agilent J&W, Santa Clara, CA, USA) at splitless mode, the flow rate of the carrier gas (99.999% helium) was 1 mL/min, the temperature of GC injector was maintained at 250 °C, the oven temperature program was started at 40 °C, held for 1 min, and increased at rate of 3 °C/min to 200 °C, ending with an isothermal period at 200 °C for 27 min. For MS detection, the ion source and interface were both programmed at 230 °C, electron-impact ionization at 70 eV, and acquisition range from 29–450 amu at scan rate of 1.5 scan/s. The volatile compounds were identified by comparison of the linear retention indices (RIS), the volatiles mass spectra fragmentation patterns with the mass data of the corresponding compounds obtained from the National Institute of Standards and Technology MS Library NIST.14. Linear RIs of the volatile components were determined relative to the retention times of a series of n-alkanes (C8–C40). Volatile compounds with mass spectral match factors over 70 and RIs matched the values in NIST.14 library were considered to be identified.

MSD ChemStation is the program used for integration. The automatic ChemStation parameters were set as follows: initial area reject 0, initial peak width 0.020, shoulder detection off, initial threshold 16.5. To qualitatively estimate the development of the volatile compounds throughout the dehydration process, the peak area of each volatile was compared against the peak area of internal standard (IS: (s)-(+)-2-octanol) using a response factor of 1. Obviously, the use of a single standard does not provide accurate quantification, but it allows the data to be normalized for comparative purposes, and so the changes in the proportion of aroma components that occur during the dehydration process are monitored. All the analyses were performed in triplicate.

### 2.4. Amino Acids Analysis

An automated amino analyzer (HITACHI L-8900, Hitachi Limited, Tokyo, Japan) was employed to quantify the amino acids [21]. The amino acid content in each sample was calculated by comparing each of the peak area with the standard amino acid. The analysis was performed in triplicate. For detailed analysis procedures, please refer to the Supplementary Materials.

### 2.5. Sugar Analysis

The sugar compositions of the samples were determined using a high-performance ion chromatography (HPIC) method adopted from Shi et al. (2019) [22] with slight modifications. All assays were performed in triplicate. For detailed procedures, please refer to the Supplementary Material.

### 2.6. Statistical Analysis

Significance differences in the volatile component profiles of the samples were tested by one-way analysis of variance (ANOVA) using SPSS 20.0 (SPSS Inc., Chicago, IL, USA). Principle component analysis (PCA) was performed using Simca-P 11.5 software (Umetrics AB, Umea, Sweden). The correlations between the identified volatile compounds and the precursors were assessed by PLSR using the Unscrambler X9.7 (CAMO ASA, Oslo, Norway). The heat map of main volatile compounds during the NCS processing was generated using Morpheus tools [23], and Venn plot analysis was performed using the OmicShare tools [24].

## 3. Results and Discussion

### 3.1. Composition of Volatile Compounds

GC-MS was used to determine the volatile components in the samples collected from the dehydration stages, and typical 3D waterfall total ion chromatograms of volatile compositions of the samples are shown in Supplementary Figure S1. Peak alignment followed by mass spectra fragmentation patterns and retention index (RI) comparison, a total of 111 volatile compounds (Supplementary Table S1) were tentatively identified, including 19 aldehydes, 10 hydrocarbons, 10 esters, 13 alcohols, 17 ketones, 13 oxygen heterocyclic compounds, nine nitrogen heterocyclic compounds, five phenols, five acids, two sulfur compounds, two terpene compounds, and six other compounds.

Prior to dehydration, 57 volatile compounds were identified in S-0 (S-t, where t represents the time when an aliquot was withdrawn from 0 to 240 min), and as dehydration proceeded the number of volatile compounds reduced to 22 at S-180 when the juice Brix was ~65 °Bx (i.e., syrup) before rising to 87 with S-240, the final NCS product (Supplementary Table S1).

**Figure 1:**
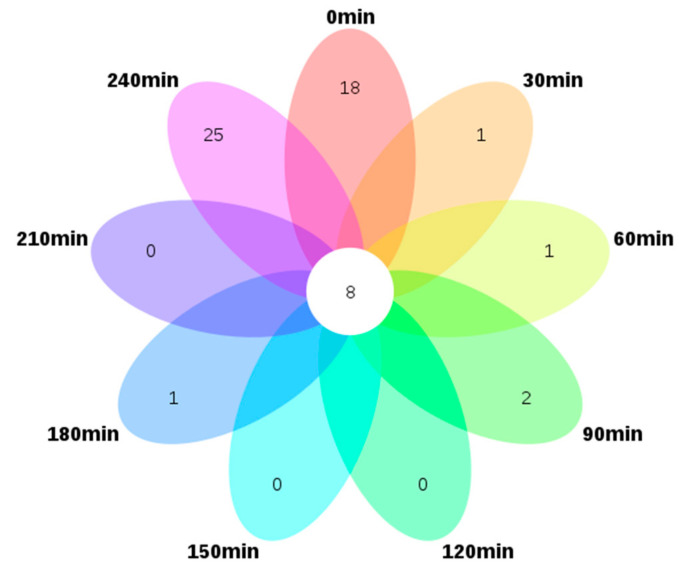
Venn diagram of 111 differential volatile compounds in nine interval samples from the NCS (non-centrifugal sugar) formation process. (Outer circle represents the volatile compounds that exist only in each sample, and central circle represents the shared volatile compounds that exist in all samples).

To visualize the relationship among the samples collected at various intervals (nine in total), a Venn plot was drawn based on the 111 identified volatile compounds. This showed the compositional variation of the volatile compounds with processing time. Interestingly, as shown in Figure 1, the nine samples only shared eight volatile compounds, namely dimethyl sulfide, 3-methyl-butanal, 2-octanone, nonanal, 2-ethyl-1-hexanol, dimethyl sulfoxide, 2,4-di-tert-butylphenol, and 2-pentyl-furan, indicating these compounds persisted throughout the dehydration process. Sample S-240 possesses the most unique volatile compounds, 25, followed by S-0 which has 18 unique species. Only one, one, two, and one unique constituent were detected in S-30, S-60, S-90, and S-180, i.e., eugenol, 3-hydroxy-2,2,4-trimethylpentyl 2-methyl-propanoate, 2-pentadecanone and nonanoic acid, and benzyl alcohol, respectively, whereas S-120, S-150 and S-210 shared all the constituents with the other interval samples.

### 3.2. Principal Component Analysis (PCA) of Volatile Profiles during the Dehydration Process

**Figure 2:**
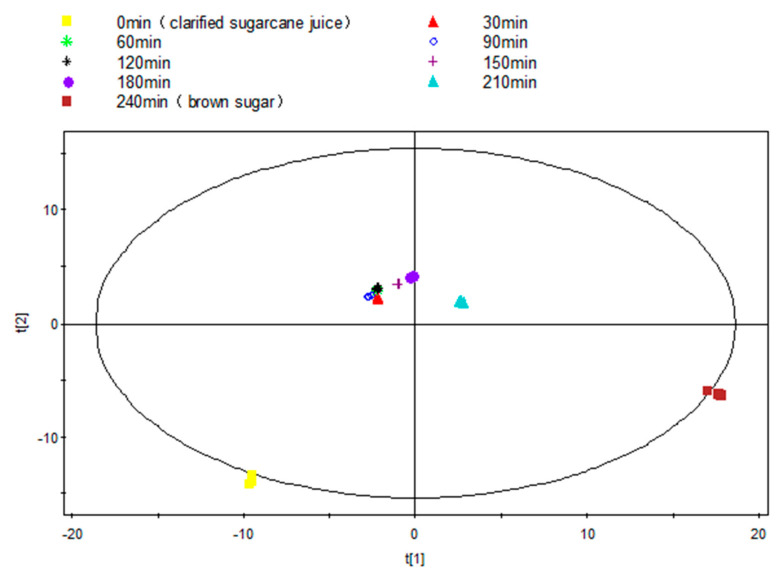
PCA score plot (R^2^X = 0.977, Q^2^ = 0.881) of interval samples from the NCS formation process over time.

As an overview of the profile of volatile compounds during the entire dehydration process, the raw peak table was statistically analyzed using unsupervised PCA. The score plot clearly revealed stepwise alterations and distinct differences of volatile compounds from S-0 to S-240 (Figure 2). An obvious difference was observed between S-0 and S-30, and this could be due to the evaporation loss of some volatile aldehydes, alcohols etc., arising from the increased temperature. For example, aldehydes including 2,4-decadienal, 2,4-nonadienal, octanal and 3-methyl-butanal, and alcohols including1-octen-3-ol existed in fresh cane juice, whereas all these aldehyde contents experienced a significant drop from S-0 to S-30.

While S-30 to S-120 clustered, which perhaps indicates a relatively stable volatile profile with no formation of new volatile compounds during this period, water evaporation is the dominant activity at such a relatively lower boiling temperature (~90 °C, 225 Torr). Further volatile profiles development was gradually observed from S-120 to S-180, obviously the concentration typical sulfur containing compound dimethyl sulfide increased eight times during this period, which could significantly contribute to the characteristic aroma. The most significant visible profile variation was observed from S-180 to S-210, with a continuous development of sulfur containing compounds from 26.12 to 46.73 µg/g, and aldehydes from 3.73 to 22.72 µg/g, ketones from 11.81 to 24.38 µg/g, heterocyclic oxygen compounds from 6.58 to 20.27 µg/g, and the initiative formation of nitrogen heterocyclic compounds, such as 1-(1-H-pyrrolo2-yl)-ethanone (0 to 0.54 µg/g) and 2,6-dimethyl-pyrazine (from 0 to 0.58 µg/g).

At the final dehydration stage, i.e., from S-210 to S-240, the profiles completely changed with significant increases in oxygen heterocyclic compounds (20.27 to 108.11 µg/g) and nitrogen heterocyclic compounds (1.12 to 12.27 µg/g). This provides a clear indication of the further development of these compounds and the reduction in the reactive aldehydes and ketones.

### 3.3. Hierarchical Cluster Analysis (HCA) and Volatile Profiles Development

**Figure 3:**
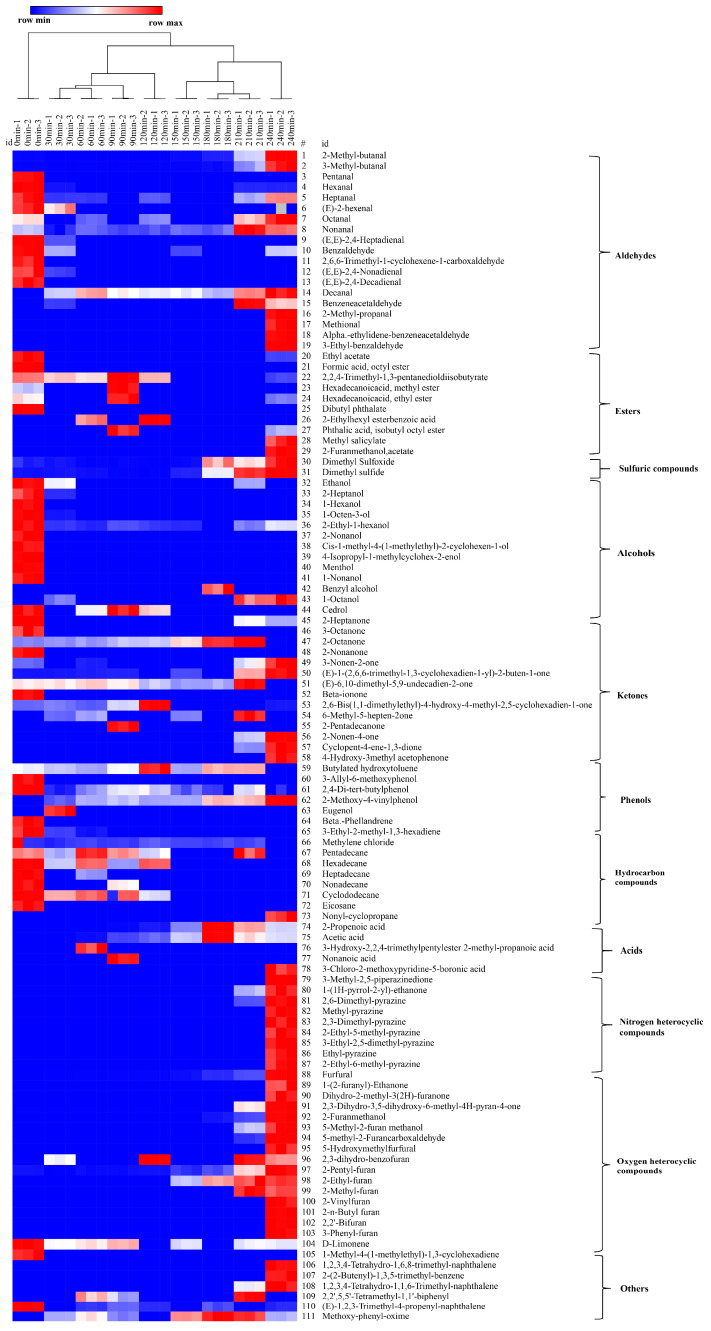
Dendrogram obtained from HCA (Hierarchical Cluster Analysis) of the 111 identified volatile compounds during the dehydration of sugarcane juice to NCS process. (The colour box for each compound indicates the abundance of the compound, the red colour annotates higher abundance while the blue colour annotates the lower abundance).

**Figure 4:**
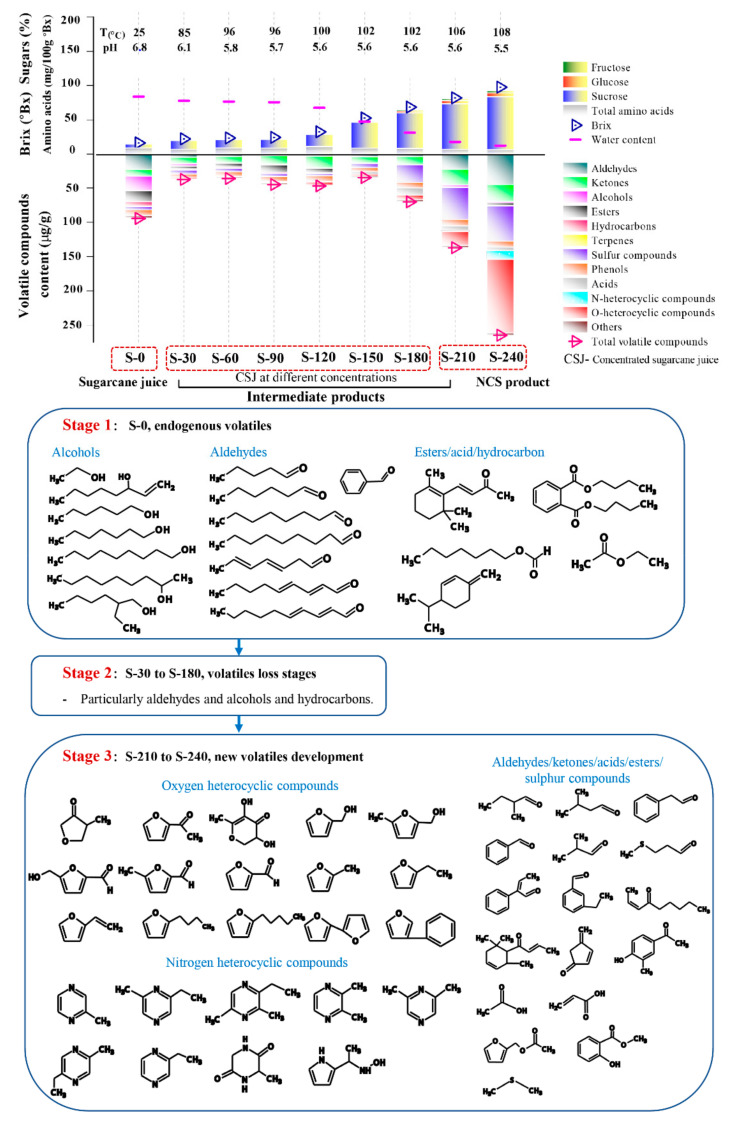
Changes of Brix, sucrose, glucose, fructose and water content at different stages during the dehydration of sugarcane juice to NCS products, and the consequent dynamic changes of volatile compounds throughout the process.

The dendrogram obtained from the HCA analysis was used to further differentiate the various dehydration stages (Figure 3). The aroma profiles of the nine samples were split into two big branches, of which branch 1 (S-0) does not cross over with any other eight samples, while S-30 to S-240 were under branch 2. However, among the eight samples, further differential analysis was conducted, whereby S-30 to S-120 were attributed to one subclass, and S-150 to S-240 assigned to another subclass. Each of the subclasses was further divided into another two branches, S-30 to S-90, S-120 and S150–210, S240. Apparently, most of the endogenous volatiles from sugarcane juice disappeared within the first 30 min, so the evaporation or further consumption of the aldehydes in the consequent Maillard reaction could be responsible for the phenomenon. The aroma of NCS developed thereafter, especially from S-150 to S-240, during which the brix increased from 45 to 98 °Bx, and the boiling temperature varied from 100 to 108 °C, while the pH decreased from 5.64 to 5.50. An overall dynamic change of the volatile compounds over the dehydration process is shown in Figure 4. Dynamic variations of typical volatile compound classes at each of the nine time intervals are discussed in the following section.

#### 3.3.1. Changes in the Concentration Profiles of Aldehydes

As shown in Figure 4, the highest aldehyde concentration was found in S-240 (45.29 µg/g), followed by S-0 (23.01 µg/g) and S-210 (22.73 µg/g). A significant variation of the aldehyde profiles was found with S-0 and S-240 (Figure 3), whereas 2-methyl-butanal (S-240, 12.07 µg/g), 3-methyl-butanal (S-240, 14.42 µg/g) with a typical chocolate and bready odor, exist in both S-0 and S-240 with their concentration developed ~100 times throughout the dehydration process. Further, 5-methyl-2-furancarboxaldehyde (caramel odor, 9.46 µg/g in S-240), 5-hydroxymethylfurfural (buttery and caramel odor, 1.52 µg/g in S-240), 2-methyl-propanal (fresh aldehydic odor, 3.55 µg/g in S-240), benzeneacetaldehyde (sweet odor, 3.47 µg/g in S-240), α-ethylidene-benzeneacetaldehyde (powdery and cocoa odor, 0.62 µg/g in S-240), 3-ethyl-benzaldehyde (bitter almond odor, 0.51 µg/g in S-240), methional (bready odor, 0.38 µg/g in S-240), emerged in the post stage of the dehydration process. Aldehydes could be generated from unsaturated fatty acids [25] and could also be derived from amino acids via Strecker degradation at a higher temperature, such as 3-methyl-butanal, 2-methyl-butanal, and 2-methyl-propanal, which could be derived from leucine, isoleucine, and valine [26]. Methional could be derived from methionine [17], and benzaldehyde could be derived from phenylalanine [27]. Meanwhile, 5-methyl-2-furancarboxaaldehyde could be derived from glucose through a series of reactions i.e., Amadori rearrangement, enolization, and cyclization [17]. Aldehydes are aroma-active compounds in both cane juice and NCS, and could also participate in the aldol condensation reactions and contribute to the formation melanoidin polymers that impact on the colour of the final product [6].

#### 3.3.2. Changes in the Concentration Profiles of Heterocyclic Compounds

Heterocyclic compounds containing the heteroatoms’ oxygen and nitrogen were by far most predominant volatile compounds accounting for 160.5 µg/g in S-240 (Figure 4). The species and overall intensity increased significantly (*p* < 0.05) at the post stage of the dehydration process, particularly furans produced by the thermal breakdown of sugars and pyrazines derived from the amino acids’ thermal degradation, both of which indicate the occurrence of the Maillard reaction [28]. These MRPs are flavor enhancing, providing typical earthy, peanut, and roasted odors to NCS [10].

Since the formation of furans only occurred at high temperature (>100 °C), volatile furans were mostly detected in the post-stages of the dehydration process, except furfural (chocolate odor), 2,3-dihydrobenzofuran, and 2-pentyl-furan (earthy, fruity odor), which were detected in the initial stages. Further, 2-furanmethanol that has bready odor was detected from S-180 and experienced a sharp increase towards the end (S-180 to S-240, 0.54 to 9.92 µg/g), which contributed to the overall NCS aroma. Meanwhile, 5-methyl-2-furanmethanol, 2,3-dihydro-3,5-dihydroxy-6-methyl-4H-pyran-4-one (DDMP), and 2-methyl-furan were all detected from S-210 (S-210 to S-240, 0.83 to 2.10, 1.81 to 3.23, and 0.30 to 0.27 µg/g, respectively) which have pleasant bready, sweet-caramel, and chocolate characteristics, respectively; 5-hydroxyfurfural (S-240, 1.52 µg/g, buttery and caramel odor) and dihydro-2-methyl-3(2H)-furanone (S-240, 0.1 µg/g, caramel odor) were identified towards the end of the dehydration process. Moreover, 2-vinyl-furan (S-240, 0.21 µg/g), 2-n-butyl-furan (S-240, 0.54 µg/g), 2,2′-bifuran (S-240, 3.98 µg/g), and 3-phenyl-furan (S-240, 4.07 µg/g) were also detected in the final dehydration process and were good alternatives of spicy odor to complement the predominant sweet-caramel odor.

Pyrazines, including 2,6-dimethyl-pyrazine (roasted nutty odor), 2-methyl-pyrazine (nutty chocolate odor), 2,3-dimethyl-pyrazine (nutty and peanut butter odor), 2-ethyl-5-methyl-pyrazine (nutty and roasted coffee odor), 3-ethyl-2,5-dimethyl-pyrazine (nutty roasted odor), ethyl-pyrazine (nutty odor), and 2-ethyl-6-methyl-pyrazine (nutty odor), were all identified in S-240 (content range from 0.46 to 3.55 µg/g), while cocoa roasted odor scented 2,6-dimethyl-pyrazine was detected from S-210, and developed obviously towards the end (3.55 µg/g). Overall, the aroma contribution of the formed heterocyclic compounds to NCS are similar to previous studies [6,10,29].

#### 3.3.3. Changes in the Concentration Profiles of Ketones

Ketones are the main sugar-derived compounds together with furans, and are generated at a relatively lower temperature, and so were identified throughout the heating and dehydration process. Seventeen ketones were found as differential aroma compounds, as shown in Figure 3 and Supplementary Table S1. Ketone concentration had a slight increasing tendency throughout the process (Figure 4), which is consistent with the results of Asikin et al. (2016) [10]. S-210 and S-240 have higher concentrations of ketones than the other samples. Among those identified differential ketones, immature apple scented 2-octanone was the only ketone that existed throughout the process, and the concentration almost tripled from S-0 to S-210 (4.07 to 11.70 µg/g), followed by a remarkable decrease in the final 30 min (S-240, 1.47 µg/g). E-6,10-dimethyl-5,9-undecadien-2-one, which presents a floral odor was another ketone that had a relatively stable concentration from S-0 to S-210 and disappeared at S-240. Moreover, E-1-(2,6,6-cyclohexadien-1-yl)-2-buten-1-one, that has a low aroma threshold value, underwent a significant increase at the post stage (S-210 to S-240, 3.23 to 4.67 µg/g) and could be acting as an aroma-active compound for NCS. Meanwhile, 2,6-bis(1,1-dimethyl)-4-hydroxy-4-methyl-2,5-cyclohexadien-1-one was identified in the initial stage and increased to the maximum value at S-120 (10.66 µg/g), followed by sharp decrease to zero at S-150, and then formed in the final product in a very small amount (0.6 µg/g). These fluctuations could be due to their roles in the Maillard reaction as they are derived from sugars through Amadori rearrangement, and continually participated in the reaction with amino compounds forming MRPs [30]. Finally, 2-nonen-4-one, cyclopent-4-ene-1,3-dione are the two ketones developed in the post dehydration stages, and their production has been reported to be related to glucose [31,32].

#### 3.3.4. Changes in the Concentration Profiles of Alcohols

Previous studies showed that alcohols represent the major volatile components in aroma performance [16,33]. As shown Figure 3, most of the alcohols were identified in the initial stage, i.e., S-0, and sharply decreased throughout the dehydration stage. Indeed, 1-octen-3-ol, an earthy odor compound, has the highest content (5.79 µg/g) in sugarcane juice, followed by ethanol (5.23 µg/g), and disappeared after 30 min. Meanwhile, green-scented 2-ethyl-1-hexanol was the only alcohol that existed in most of the samples except S-180, the final concentration was 1.79 µg/g in S-240. Waxy odor scented 1-octanol was the other alcohol detected in the final sugar product.

#### 3.3.5. Changes in the Concentration Profiles of Esters

Esters are generally known to the fruity and floral flavor of foods derived from esterification of fatty acids with alcohols [34]. As shown in Figure 3 and Figure 4, most of the esters were identified in the early-stage samples (S-0 to S-120). Typically, 2,2,4-trimethyl-1,3-pentanedioldiisobutyrate (no odor) has the highest content (5.60 µg/g) in S-0 followed by dibutyl phthalate (faint odor, 5.14 µg/g), ethyl acetate (etherial, fruity and rum-like odor, 2.67 µg/g), and octyl formate (fruity odor, 1.31 µg/g). It is worth noting that octyl formate has recently been identified as volatile biomarker in Jintao (*A. chinensis*), providing a pleasant odor [35], and ethyl acetate has been recognized as a vital flavor component of sugarcane juice [36]. Methyl salicylate (minty odor) and acetate (banana-like odor) were the esters that developed only in S-240, with concentrations of 0.22 and 1.15 µg/g, respectively. Methyl hexadecanoate and ethyl hexadecanoate with waxy, fruity, and creamy odor esters found in *Castanopsis fissa* wood [37] were both identified in S-0 and S-90 and absent in other intervals, except the latter, which developed in a tiny amount in the final NCS product (0.29 µg/g).

#### 3.3.6. Changes in the Concentration Profiles of Hydrocarbons and Terpene Hydrocarbons

Ten hydrocarbon compounds were identified in among the nine samples, as shown in Figure 3 and Figure 4. Nevertheless, only one alkane, nonyl-cyclopropane (0.03 µg/g) was detected in final product S-240, which supports previous reports in the literature [4,6,10]. Most of the hydrocarbons were found in the initial clarified juice sample S-0, their intensity attenuated gradually thereafter with some fluctuations, among which the minty scented *β*-phellandrene (0.32 µg/g) was the only monoterpene hydrocarbon detected in the S-0. At the initial stage, 3-ethyl-2-methyl-1,3-hexadiene had the highest concentration (2.29 µg/g) and went down to zero at S-90. However, the hydrocarbons identified in fresh cane juice were not classified as an aroma component as they have high odor threshold values [13,14].

D-limonene was the only terpene hydrocarbon that was detected throughout the heating and dehydration process, except in S-180, and was present in the final product (S-240, 0.2 µg/g), presenting a floral-like odor contribution to the NCS aroma.

#### 3.3.7. Changes in the Concentration Profiles of Sulfur-Derived Compounds, Phenols, Acids and Others

Sulfur compounds, phenols, acids, and naphthalene also contribute to the overall aroma. The sulfur compounds dimethyl sulfide and dimethyl sulfoxide that have been classed as heat-derived sulfur compounds [38,39] were detected in the samples, of which the dimethyl sulfide has been identified in fresh NCS sample accounting for 11.96% of the volatile flavor components [6]. In the dehydration process, it experienced a 10-fold increase with the content from S-0 to S-240 (5.11 to 50.49 µg/g), which could originate from the Strecker degradation of the cysteine and methionine that existed in the system [27,40], acting as an off-odor generator due to the extremely low sensory threshold values. Meanwhile, dimethyl sulfoxide decreased from S-0 to S-150, and then increased slightly from S-180 to the final product (0.94 µg/g).

Five (5) phenols were identified, of which butylated-hydroxy-toluene presented in S-0 to S-210 and disappeared in the final product. Throughout the process, 2,4-di-tert-butylphenol, which has previously identified in sugarcane juice, grape berries, and pomelo juice [13,41,42], was the only phenol detected, and had its highest concentration in S-0 but then showed an overall decreasing trend (S-0 to S-240, 6.70 to 2.65 µg/g). Then, 2-methoxy-4-vinylphenol, a woody odor phenol identified in the fresh NCS [6], was also identified from S-30 to S-240 and showed an increasing trend (from 1.05 to 5.92 µg/g). Spicy odor scented eugenol was only identified in S-30. In addition to the aroma supplementary characteristics, these phenols also contribute to the antimicrobial properties [43].

Three types of acids that were detected in sample S-30 to S-240, while were absent in the initial juice, indicating that no microbial or chemical degradation occurred prior to the dehydration process [44,45]. Among the detected acids, acetic acid, which provides an acidic odor, was detected in S-60 and progressively decreased in the other samples. Further, 2-propenoic acid was the other dominant acid identified and experienced an increasing trend from S60 to S-180 (0.14 to 6.06 µg/g), followed by a decreasing trend towards the end (S-240, 2.54 µg/g). The acid concentration in the NCS produced in this study was much lower than the values reported in previous studies [6,10,16,33]. This could be attributed to the relatively lower temperature used, no microbial or chemical degradation in the initial sugarcane juice, and reduced amounts of fine fibres by membrane filtration.

Six (6) naphthalene compounds, including 1,2,3,4-tetrahydro-1,6,8-trimethyl-naphthalene, 2-(2-butenyl)-1,3,5-trimethyl-benzene, 1,2,3,4-tetrahydro-1,1,6-trimethyl-naphthalene, 2,2′,5,5′-tetramethyl-1,1′-biphenyl, (E)-1,2,3-trimethyl-4-propenyl-naphthalene, and methoxy-phenyl-oxime, that have not been reported as the aroma profiles in NCS, were identified in this study, and the first three of them are only developed at the very end of the process. The concentration of the other three fluctuated through the process, in which (E)-1,2,3-trimethyl-4-propenyl-naphthalene was the only compound detected in initial sugarcane juice S-0.

### 3.4. Volatile Compounds Formation and Their Precursors

As observed from the overall compositional profiles of the intermediates during dehydration of membrane-clarified sugarcane juice (Figure 4), S-0 had a relatively high content of volatile compounds, among which aldehydes, alcohols, hydrocarbons, and esters are the four major components that form the typical aroma present in fresh cane juice. As shown in Figure 4 Stage1, aldehydes and alcohols present in sugarcane juice are mainly long chain fatty aldehydes and alcohols. Meanwhile, from S-30 to S-180 (Figure 4 Stage 2), there is a significant loss of these endogenous volatiles, which could be due to the evaporation caused by the increased temperature and vacuum applied to the system. The development of the aroma compounds present in NCS mainly occurred at the post dehydration stages from S-210 to S-240 (Figure 4 Stage 3), during which the temperature was 106–108 °C and pH was ~5.50. Aldehydes, ketones, oxygen heterocyclic compounds, typically furans, and nitrogen heterocyclic, typically pyrazines, were found to predominate, which are associated with both caramelization and Maillard reactions. Sugars are the dominate components in sugarcane juice, and represent a source for caramelization, while the chemistry underlying the Maillard reaction is a complex reaction between the carbonyl group of reducing sugars and amine group of free amino acid, peptides, and proteins [46]. A reasonable number of proteins and free amino acids (FAA) are present in juice which could provide reactants with sufficient basicity for the reaction to occur.

Partial least squares regression (PLSR) analysis was used to determine the correlation between each volatile species and each of the FAA and sugars (Supplementary Section S2.1 and Figure S2a). PLSR analysis confirmed that volatiles including aldehydes, sulphur compounds, acids, oxygen, and nitrogen heterocyclic compounds formation were also positively associated with glucose and fructose, while negatively associated with sucrose, indicating that sucrose was not directly involved in the formation of volatile compounds. However, as sucrose (~19.78%) is the dominant sugar in sugarcane juice, is readily inverted to glucose and fructose under acidic conditions (6.8 to 5.5) and high temperature (85 to 108 °C). This is shown with the increased glucose and fructose concentrations found at the end of the dehydration process (S-0 to S-240, 7.8 to 67.9 and 11.8 to 36.4 mg/kg·°Bx, respectively).

**Figure 5:**
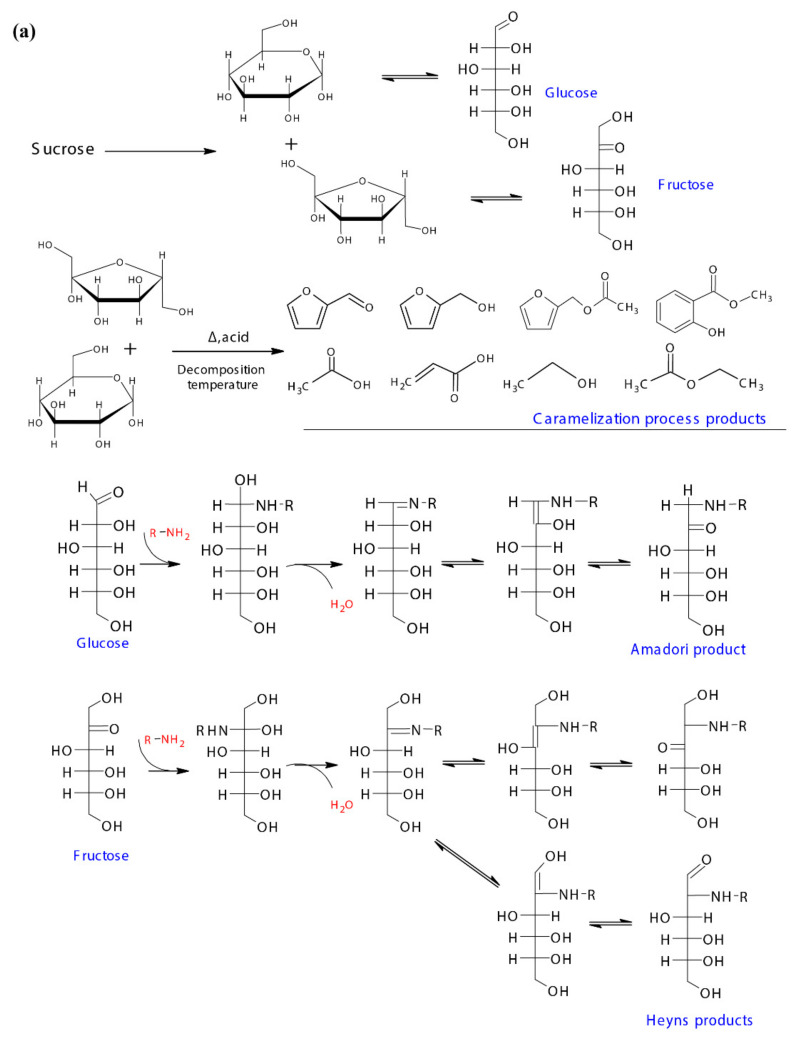

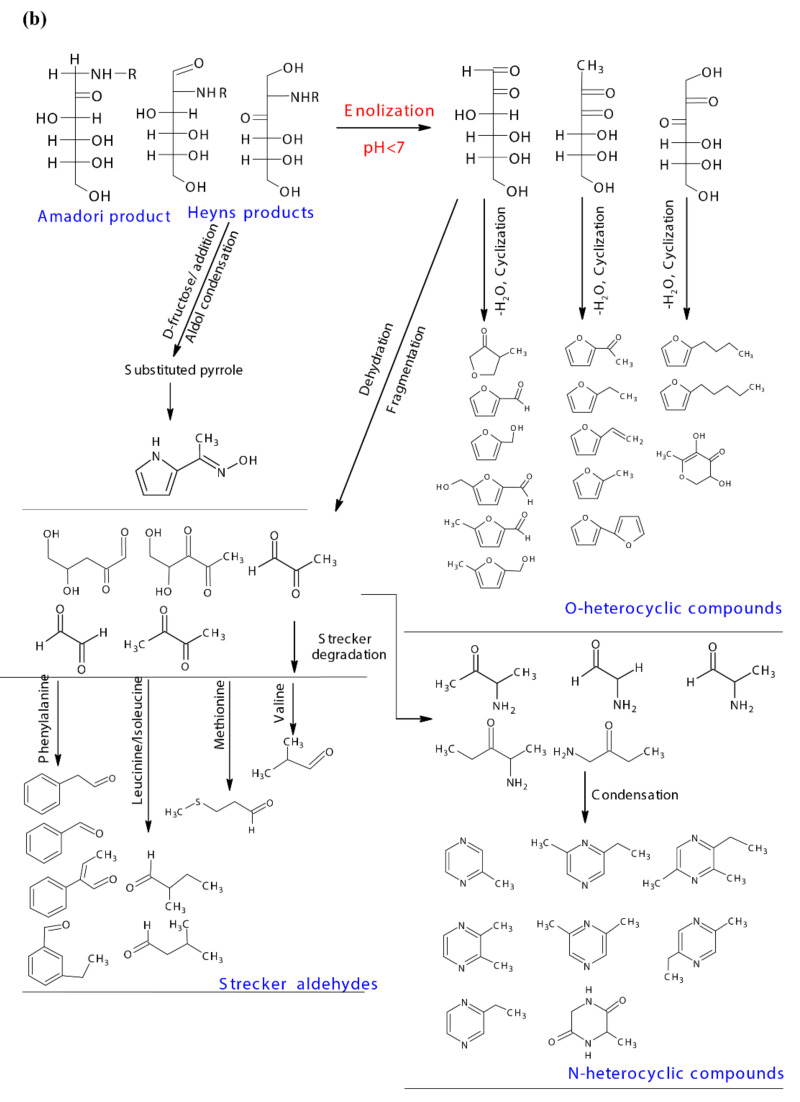
(**a**) Sucrose chemical and thermal inversion process, the possible caramelization reaction pathway of mono sugars, and the initiation of mono sugars Maillard reaction pathways, and (**b**) the possible Maillard reaction pathways for the typical volatiles formed in the dehydration process.

A possible reaction pathway of the inversion of sucrose to mono sugars i.e., glucose and fructose, and their further participation in the caramelization and Maillard reaction are shown in Figure 5a,b. The mono sugars could cyclize at the relatively high temperature to produce aromatics and furans, and produce acids, aldehydes, alcohols and other compounds through oxidation and reduction [47]. Furfural, 2-furanmethanol, 2-furanmethanol acetate, methyl salicylate, acetic acid, and 2-propenoic acid that developed in the post dehydration process could be caramelization products, while ethanol and ethyl acetate were identified in fresh sugarcane juice indicated the caramelization occurred under the higher temperature in the membrane filtration process (Figure 5a). The participation of glucose and fructose in the Maillard reaction was initiated by the condensation of their carbonyl groups with amino groups from amino acids/peptides/proteins, and Schiff bases were formed in this step, which then undergo Amadori and Heyns rearrangements respectively. The Amadori product and Heyns products undergone enolization, deamination process, forming deoxy-dicarbonyl sugar derivatives [48]. As the pH of the current reaction system was <7, only 1,2-enaminol process occurred for the Amadori product, forming the 3-deoxyglucosone, while for Heyns products, both 3-deoxyfructosone and 4-deoxyfructosone were formed. The deoxy-dicarbonyl sugar derivatives underwent further dehydration and cyclization processes, forming a series of oxygen heterocyclic compounds, such as furan compounds and furanones (Figure 5b). Additionally, they could also undergo dehydration, and fragmentation, giving rise to products containing one or more carbonyl groups [49], which could be further involved in the consequent Strecker degradation processes. A possible addition reaction between Heyns products and D-fructose was proposed by Dills Jr. (1993) [48], with further aldol condensation and dehydration, forming the substituted pyrrole. Thus, the 1-(1H-pyrrol-2-yl)-ethanone developed from S-210 towards the end could be the derivative of the substituted pyrrole.

PLSR analysis also indicated that all the FAA were positively correlated to the formation of aldehydes, sulfur compounds, acids, oxygen, and nitrogen heterocyclic compounds, while negatively correlated to esters and hydrocarbons (Figure S2b). Total FAA content in the system experienced an overall decreasing trend from 87.69 at the beginning to 62.62 mg/kg·°Bx towards the end (Table 1). However, each FAA content fluctuated from S-0 to S120, followed by a decreasing trend thereafter (Supplementary Figure S3), the fluctuation could be due to the consumption of FAA in the early stages of the Maillard reaction (a relative mild heating condition), forming Amadori products [50], and the release of amino groups from the furfural-based Schiff base intermediates that occurred at a higher temperature among the dehydration process [51]. Meanwhile, the decreasing trend from S150 to S-240 could be due to the consumption of amino acids in the Strecker degradation process, forming Strecker aldehydes and amino containing carbonyl compounds. As shown in Figure 4 Stage 3, Strecker aldehydes developed in the process were significantly differed from that in fresh cane juice, most of them are short chain aldehydes. The possible reaction pathway of the typical Strecker aldehydes formed in the dehydration process are shown in Figure 5b. The amino containing carbonyl products further react with the amino groups, followed by condensation or polymerization process, forming pyrazines and melanoidins that contribute to the flavor and browning appearance of the products, respectively [52]. The possible reaction pathway of the typical nitrogen heterocyclic compound pyrazines identified from S-210 to S-240 are shown in Figure 5b.

In addition to aldehydes, the heterocyclic compounds containing the heteratoms’ oxygen and nitrogen, sulfur compounds, including dimethyl sulfide and dimethyl sulfoxide, were also increased significantly in the post stages (S-180 to S-240). The concentration of the furfural, dimethyl sulfide and dimethyl sulfoxide are particularly high, and it would be beneficial to reduce or eliminate them from NCS without affecting the aroma characteristic but still maintain health and nutritional promoting properties. The boiling point of some of these volatile compounds, e.g., dimethyl sulfide (37 °C), is low and so the composition of the aroma compounds of NCS will vary during storage, thereby impacting on the sugar odor. In this regard, the dehydrating process should be re-designed and may involve changing the reaction temperature and time profiles.

## 4. Conclusions

In the present study, the dynamic changes of the various types of volatile compounds and their precursors during the dehydration of membrane-clarified juice was examined. Results from Venn plotting, PCA, and HCA analyses revealed significant differences of the volatile profiles and aroma characteristics among sugarcane juice, samples of concentrated juice, and the NCS product. The proportions of MRPs, including aldehydes, ketones, and heterocyclic compounds (furans and pyrazines), that give the characteristic aroma to NCS increased significantly after long reaction times. The distinction in the proportions of the volatile aroma compounds developed in the dehydration of sugarcane juice to NCS could provide a basis for the design of a specific aroma for NCS based food and beverage products. The findings of this study have also indicated that, to obtain a good balance between bioactive compounds and odor active volatile compounds in NCS, a detailed dehydration kinetic study on NCS is needed.

## Figures and Tables

**Table 1:** Changes of free amino acids concentration during the dehydration of sugarcane juice to NCS (non-centrifugal sugar) products.

Amino Acids	Concentration (mg/100 g·°Bx)									Consumption (%)
	S-0	S-30	S-60	S-90	S-120	S-150	S-180	S-210	S-240	
Asp	0.903 ^b^	0.702 ^c^	0.921 ^b^	0.898 ^b^	1.185 ^a^	0.930 ^b^	0.899 ^b^	0.881 ^b^	0.874 ^b^	3.21
Thr	0.184 ^b^	0.143 ^d^	0.184 ^b^	0.180 ^b c^	0.230 ^a^	0.176 ^b c^	0.166 ^c^	0.149 ^d^	0.138 ^d^	25
Ser	0.501 ^a,b^	0.385 ^b^	0.495 ^a,b^	0.484 ^b^	0.626 ^a^	0.487 ^b^	0.462 ^b^	0.407 ^b^	0.389 ^b^	22.36
Glu	5.196 ^b^	4.075 ^c,d,e^	5.227 ^b^	5.101 ^b,c^	6.554 ^a^	5.008 ^b,c^	4.664 ^b,c,d^	3.927 ^d,e^	3.573 ^e^	31.24
Gly	0.038 ^a,b^	0.026 ^b^	0.035 ^a,b^	0.035 ^a,b^	0.049 ^a^	0.039 ^a,b^	0.035 ^a,b^	0.031 ^a,b^	0.027 ^b^	28.95
Ala	0.393 ^a,b^	0.305 ^b^	0.391 ^a,b^	0.383 ^a,b^	0.500 ^a^	0.388 ^a,b^	0.367 ^b^	0.334 ^b^	0.301 ^b^	23.41
Cys	0.008 ^a,b^	0.006 ^a,b,c^	0.007 ^a,b^	0.007 ^a,b^	0.012 ^a^	0.009 ^a,b^	0.006 ^a,b,c^	0.003 ^b,c^	0.000 ^d^	100
Val	0.264 ^b^	0.205 ^b,c^	0.265 ^b^	0.259 ^b^	0.347 ^a^	0.268 ^b^	0.248 ^b^	0.216 ^b,c^	0.183 ^c^	30.68
Met	0.033 ^c,d^	0.024 ^d^	0.037 ^b,c,d^	0.036 ^b,c,d^	0.065 ^a^	0.053 ^a,b^	0.045 ^b,c^	0.036 ^b,c,d^	0.028 ^c,d^	15.15
Ile	0.137 ^a,b^	0.108 ^b^	0.143 ^a,b^	0.138 ^a,b^	0.192 ^a^	0.151 ^a,b^	0.137 ^a,b^	0.113 ^b^	0.089 ^b^	35.04
Leu	0.103 ^a^	0.083 ^a^	0.113 ^a^	0.110 ^a^	0.155 ^a^	0.123 ^a^	0.112 ^a^	0.109 ^a^	0.085 ^a^	17.48
Tyr	0.143 ^a,b^	0.115 ^b^	0.150 ^a,b^	0.146 ^a,b^	0.200 ^a^	0.157 ^a,b^	0.143 ^a,b^	0.133 ^b^	0.115 ^b^	19.58
Phe	0.150 ^a,b^	0.124 ^b^	0.163 ^a,b^	0.153 ^a,b^	0.205 ^a^	0.162 ^a,b^	0.147 ^a,b^	0.123 ^b^	0.096 ^b^	36
Lys	0.106 ^a^	0.080 ^a^	0.106 ^a^	0.098 ^a^	0.126 ^a^	0.097 ^a^	0.094 ^a^	0.058 ^a^	0.050 ^a^	52.83
His	0.182 ^a^	0.151 ^a^	0.211 ^a^	0.168 ^a^	0.211 ^a^	0.156 ^a^	0.154 ^a^	0.085 ^b^	0.084 ^b^	53.85
Arg	0.156 ^b^	0.122 ^c^	0.161 ^b^	0.157 ^b^	0.205 ^a^	0.159 ^b^	0.149 ^b^	0.119 ^c^	0.071 ^d^	54.49
Hypro	0.255 ^a^	0.203 ^a,b^	0.245 ^a^	0.253 ^a^	0.272 ^a^	0.204 ^a,b^	0.192 ^a,b^	0.182 ^a,b^	0.136 ^b^	46.67
Pro	0.017 ^a^	0.012 ^a^	0.016 ^a^	0.022 ^a^	0.016 ^a^	0.013 ^a^	0.013 ^a^	0.013 ^a^	0.023 ^a^	−35.29
Σ	8.769 ^b^	6.869 ^e^	8.870 ^b^	8.628 ^c^	11.150 ^a^	8.590 ^c^	8.033 ^d^	6.919 ^e^	6.262 ^f^	28.59

Each value is the mean of triplicate determinations ± standard deviation and values with different lowercase within the same row are significantly different (*p* < 0.05).

## Supplementary Materials

The following are available online at https://www.mdpi.com/article/10.3390/foods10071561/s1, Figure S1: 3D waterfall total ion chromatograms of volatile compounds, Figure S2: Correlation loadings plot of volatile compounds and (a) free amino acid, and (b) sugars during NCS formation process. (Aldehydes-1, esters-2, sulphur compounds-3, alcohols-4, ketones-5, phenols-6, hydrocarbons-7, acids-8, nitrogen compounds-9, oxygen heterocyclic compounds-10, terpene compounds-11, others-12), Figure S3: Amino acids percentage variation at different stages during the dehydration of sugarcane juice to NCS products and Table S1: Volatile compounds and the relative concentrations (µg/g) identified in the interval samples during the NCS formation process using headspace-solid phase microextraction and gas chromatography–mass spectrometry (HS-SPME and GC-MS).

## References

[1] Zhu Z., Xie C., Li W., Hang F., Li K., Shi C., Doherty W.O.S. (2020). Nutritional and antioxidant properties of non-centrifugal cane sugar derived from membrane clarified juice. LWT.

[2] Harish Nayaka M.A., Sathisha U.V., Manohar M.P., Chandrashekar K.B., Dharmesh S.M. (2009). Cytoprotective and antioxidant activity studies of jaggery sugar. Food Chem..

[3] Jaffé W.R. (2012). Health Effects of Non-Centrifugal Sugar (NCS): A Review. Sugar Tech..

[4] Lee J.S., Ramalingam S., Jo I.G., Kwon Y.S., Bahuguna A., Oh Y.S., Kwon O.J., Kim M. (2018). Comparative study of the physicochemical, nutritional, and antioxidant properties of some commercial refined and non-centrifugal sugars. Food Res. Int..

[5] Seguí L., Calabuig-Jiménez L., Betoret N., Fito P. (2015). Physicochemical and antioxidant properties of non-refined sugarcane alternatives to white sugar. Int. J. Food Sci. Technol..

[6] Asikin Y., Kamiya A., Mizu M., Takara K., Tamaki H., Wada K. (2014). Changes in the physicochemical characteristics, including flavour components and Maillard reaction products, of non-centrifugal cane brown sugar during storage. Food Chem..

[7] Jaffé W.R. (2015). Nutritional and functional components of non-centrifugal cane sugar: A compilation of the data from the analytical literature. J. Food Compos. Anal..

[8] García J.M., Narváez P.C., Heredia F.J., Orjuela Á., Osorio C. (2017). Physicochemical and sensory (aroma and colour) characterisation of a non-centrifugal cane sugar (“panela”) beverage. Food Chem..

[9] Payet B., Shum Cheong Sing A., Smadja J. (2005). Assessment of antioxidant activity of cane brown sugars by ABTS and DPPH radical scavenging assays: Determination of their polyphenolic and volatile constituents. J. Agric. Food Chem..

[10] Asikin Y., Hirose N., Tamaki H., Ito S., Oku H., Wada K. (2016). Effects of different drying-solidification processes on physical properties, volatile fraction, and antioxidant activity of non-centrifugal cane brown sugar. LWT Food Sci. Technol..

[11] Naknean P., Meenune M. (2015). Impact of Clarification of Palm Sap and Processing Method on the Quality of Palm Sugar Syrup (*Borassus Flabellifer* L.). Sugar Tech..

[12] Tokitomo Y., Kobayashi A., Yamanishi T. (1984). Aroma components of fresh sugar cane juice. Agric. Biol. Chem..

[13] Wang L., Wang P., Deng W., Cai J., Chen J. (2019). Evaluation of aroma characteristics of sugarcane (*Saccharum officinarum* L.) juice using gas chromatography-mass spectrometry and electronic nose. LWT.

[14] Wang L., Deng W., Wang P., Huang W., Wu J., Zheng T., Chen J. (2020). Degradations of aroma characteristics and changes of aroma related compounds, PPO activity, and antioxidant capacity in sugarcane juice during thermal process. J. Food Sci..

[15] Weerawatanakorn M., Asikin Y., Takahashi M., Tamaki H., Wada K., Ho C.T., Chuekittisak R. (2016). Physico-chemical properties, wax composition, aroma profiles, and antioxidant activity of granulated non-centrifugal sugars from sugarcane cultivars of Thailand. J. Food Sci. Technol..

[16] Asikin Y., Takahara W., Takahashi M., Hirose N., Ito S., Wada K. (2017). Compositional and Electronic Discrimination Analyses of Taste and Aroma Profiles of Non-Centrifugal Cane Brown Sugars. Food Anal. Methods.

[17] Liu J., Wan P., Xie C., Chen D.W. (2021). Key aroma-active compounds in brown sugar and their influence on sweetness. Food Chem..

[18] Del Castillo M.D., Corzo N., Olano A. (1999). Early stages of Maillard reaction in dehydrated orange juice. J. Agric. Food Chem..

[19] Higa M., Freitas A.J., Bannwart A.C., Zemp R.J. (2009). Thermal integration of multiple effect evaporator in sugar plant. Appl. Therm. Eng..

[20] Ma C., Li J., Chen W., Wang W., Qi D., Pang S., Miao A. (2018). Study of the aroma formation and transformation during the manufacturing process of oolong tea by solid-phase micro-extraction and gas chromatography–mass spectrometry combined with chemometrics. Food Res. Int..

[21] Shim Y.S., Yoon W.J., Ha J., Seo D., Lee K.W., Lee W.Y., Kwon K., Kang T.S., Lee J.H., Kim H.J. (2013). Method validation of 16 types of structural amino acids using an automated amino acid analyzer. Food Sci. Biotechnol..

[22] Shi C., Rackemann D.W., Moghaddam L., Wei B., Li K., Lu H., Xie C., Hang F., Doherty W.O.S. (2019). Ceramic membrane filtration of factory sugarcane juice: Effect of pretreatment on permeate flux, juice quality and fouling. J. Food Eng..

[23] MorpheusSoftware. https://software.broadinstitute.org/morpheus.

[24] OmicShare Tools. http://www.omicshare.com/tools.

[25] Chen Q., Zhu Y., Dai W., Lv H., Mu B., Li P., Tan J., Ni D., Lin Z. (2019). Aroma formation and dynamic changes during white tea processing. Food Chem..

[26] Emmanuel Ohene A., Alistair P., Mark F., Angela R. (2008). Flavor formation and character in cocoa and chocolate: A critical review. Crit. Rev. Food Sci. Nutr..

[27] Rottiers H., Tzompa Sosa D.A., De Winne A., Ruales J., De Clippeleer J., De Leersnyder I., De Wever J., Everaert H., Messens K., Dewettinck K. (2019). Dynamics of volatile compounds and flavor precursors during spontaneous fermentation of fine flavor Trinitario cocoa beans. Eur. Food Res. Technol..

[28] Pihlsgård P., Larsson M., Leufvén A., Lingnert H. (2000). Volatile compounds in the production of liquid beet sugar. J. Agric. Food Chem..

[29] Asikin Y., Wada K., Imai Y., Kawamoto Y., Mizu M., Mutsuura M., Takahashi M. (2018). Compositions, taste characteristics, volatile profiles, and antioxidant activities of sweet sorghum (*Sorghum bicolor* L.) and sugarcane (*Saccharum officinarum* L.) syrups. J. Food Meas. Charact..

[30] Liu J., Liu M., He C., Song H., Chen F. (2015). Effect of thermal treatment on the flavor generation from Maillard reaction of xylose and chicken peptide. LWT Food Sci. Technol..

[31] De Schutter D. (2008). The Influence of Thermal Load during Wort Boiling on the Flavour Stability of Beer. Ph.D. Thesis.

[32] Suzuki M., Matsumoto S., Mizoguchi M., Hirata S., Takagi K., Hashimoto I., Yamano Y., Ito M., Fleischmann P., Winterhalter P. (2002). Identification of (3S, 9R)-and (3S, 9S)-Megastigma-6,7-dien-3,5,9-triol 9-O-β-D-glucopyranosides as Damascenone Progenitors in the…. Biosci. Biotechnol. Biochem..

[33] Edris A.E., Murkovic M., Siegmund B. (2007). Application of headspace-solid-phase microextraction and HPLC for the analysis of the aroma volatile components of treacle and determination of its content of 5-hydroxymethylfurfural (HMF). Food Chem..

[34] Christie W. (1993). Preparation of ester derivatives of fatty acids for chromatographic analysis. Advances in Lipid Methodology.

[35] Zhang C., Zhang Q., Zhong C., Guo M. (2019). Volatile fingerprints and biomarkers of three representative kiwifruit cultivars obtained by headspace solid-phase microextraction gas chromatography mass spectrometry and chemometrics. Food Chem..

[36] Yang H., Wang S., Yu S., Zeng X., Sun D. (2014). Characterization and semiquantitative analysis of volatile compounds in six varieties of sugarcane juice. Int. J. Food Eng..

[37] Zhang Z.F., Peng W.X., Li K.F., Ma Q.Z. (2008). Study on Medicinal Properties of Red 1% NaOH Extractives of Castanopsis Fissa Wood by GC/MS. Mater. Sci. Forum.

[38] Segurel M.A., Razungles A.J., Riou C., Trigueiro M.G., Baumes R.L. (2005). Ability of possible DMS precursors to release DMS during wine aging and in the conditions of heat-alkaline treatment. J. Agric. Food Chem..

[39] Xu Y.X., Zhang M., Fang Z.X., Sun J.C., Wang Y.Q. (2014). How to improve bayberry (*Myrica rubra* Sieb. et Zucc.) juice flavour quality: Effect of juice processing and storage on volatile compounds. Food Chem..

[40] Ahn D.U., Lee E.J., Feng X., Zhang W., Lee J.H., Jo C., Nam K. (2016). Mechanisms of volatile production from sulfur-containing amino acids by irradiation. Radiat. Phys. Chem..

[41] Miklósy É., Kerényi Z. (2004). Comparison of the volatile aroma components in noble rotted grape berries from two different locations of the Tokaj wine district in Hungary. Anal. Chim. Acta.

[42] Cheong M.W., Liu S.Q., Zhou W., Curran P., Yu B. (2012). Chemical composition and sensory profile of pomelo (*Citrus grandis* (L.) Osbeck) juice. Food Chem..

[43] Zhao C., Zeng Y., Wan M., Li R., Liang Y., Li C., Zeng Z., Chau F.T. (2009). Comparative analysis of essential oils from eight herbal medicines with pungent flavor and cool nature by GC–MS and chemometric resolution methods. J. Sep. Sci..

[44] Godshall M.A., DeLucca A.J. (1984). Acetic acid, a major volatile constituent of brown sugar: Its origin and measurement. J. Agric. Food Chem..

[45] Qureshi M.S., Bhongale S.S., Thorave A.K. (2011). Determination of organic acid impurities in lactic acid obtained by fermentation of sugarcane juice. J. Chromatogr. A.

[46] Hodge J.E. (1953). Dehydrated foods, chemistry of browning reactions in model systems. J. Agric. Food Chem..

[47] De Aguiar C.L., Rocha A.L.B., Jambassi J.R., Sampaio A. (2015). Factors Affecting Color Formation During Storage of White Crystal Sugar. Focusing Mod. Food Ind..

[48] Dills W.L. (1993). Protein fructosylation: Fructose and the Maillard reaction. Am. J. Clin. Nutr..

[49] Halford N.G., Curtis T.Y., Muttucumaru N., Postles J., Elmore J.S., Mottram D.S. (2012). The acrylamide problem: A plant and agronomic science issue. J. Exp. Bot..

[50] Huang M.G., Zhang X.M., Eric K., Abbas S., Hayat K., Liu P., Xia S.Q., Jia C.S. (2012). Inhibiting the color formation by gradient temperature-elevating Maillard reaction of soybean peptide-xylose system based on interaction of l-cysteine and Amadori compounds. J. Pept. Sci..

[51] Van Lancker F., Adams A., De Kimpe N. (2010). Formation of pyrazines in Maillard model systems of lysine-containing dipeptides. J. Agric. Food Chem..

[52] Perez-Locas C., Yaylayan V.A. (2010). The Maillard reaction and food quality deterioration. Woodhead Publishing Series in Food Science, Technology and Nutrition, Chemical Deterioration and Physical Instability of Food and Beverages.

