# Supplementary material for: Formation of Volatile and Aroma Compounds during the Dehydration of Membrane-Clarified Sugarcane Juice to Non-Centrifugal Sugar
Source: Foods. 2021 Jul 5;10(7):1561. doi: 10.3390/foods10071561 (PMC8303542; doi:10.3390/foods10071561)
Supplement: Supplementary file 1 [file foods-10-01561-s001.zip › foods-1272477-supplementary.pdf]

Supplementary Materials

Formation of Volatile and Aroma Compounds during the Dehydration of Membrane-Clarified Sugarcane Juice to Non-Centrifugal Sugar

Yanjing Ge [1], Kai Li [1,2,3], Caifeng Xie [1,2,3], Yongshi Xu [3,4], Changrong Shi [1,5,6] *, Fangxue Hang [1,2,3,*] and William O.S. Doherty [5,6]

1 College of Light Industry and Food Engineering, Guangxi University, Nanning 530004, China;
gyjpapers@163.com (Y.G.); gxlikai@gxu.edu.cn (K.L.); fcx11@163.com (C.X.)
2 Provincial and Ministerial Collaborative Innovation Center for Sugar Industry, Nanning 530004, China
3 Engineering Research Center for Sugar Industry and Comprehensive Utilization, Ministry of Education, Nanning 530004, China; xuyongsih@163.com
4 Guangxi Baiguitang Food Technology Co., Ltd., Guigang 537321, China
5 Centre for Agriculture and the Bioeconomy, Institute for Future Environments, Queensland University of Technology, Brisbane, QLD 4000, Australia; w.doherty@qut.edu.au
6 School of Mechanical, Medical and Process Engineering, Science and Engineering Faculty,
Queensland University of Technology, Brisbane, QLD 4000, Australia
* Correspondence: c.shi@qut.edu.au (C.S.); hangfx@163.com (F.H.); Tel.: +61-0731381476 (C.S.);
Tel.: +86-07713231590 (F.H.)

1. Experimental Methods

1.1. Extraction and analysis of volatile components

The volatile compounds of each sugar sample were extracted using headspace-solid phase microextraction (HS-SPME). The method was adopted from Huang (2019) with slight modifications. Approximately 0.6 g NaCl was added into a 40 mL headspace vial with 6 mL sugar solution (juice or diluted syrup/sugar), and 6 μL of 2-octanol was added as internal standard. The vial was sealed tightly and stabilized in 50 ℃ water bath for 3 min, the DVB/CAR/PDMS coated (50/30μm; 1 cm) SPME fibre (Supelco, St. Louis, MO, USA) was inserted in the headspace of the vial to absorb the volatile compounds and incubated for 50 min with agitation at 350 rpm. Finally, thermal desorption of the volatile compounds was performed in the GC-MS injection port at 250 °C for 3 min. To avoid contamination, SPME fibre was inserted in the GC injection port and aged for 30 min at 250 °C prior to use.

1.2. Amino acids analysis

An automated amino analyzer (HITACHI L-8900, Hitachi Limited, Tokyo, Japan) was employed to quantify the free amino acids (Shim *et al*., 2013). Briefly, 1.0 g of sample was dissolved in 10 mL 4% (wt/vol) sulfosalicylic acid, and kept at room temperature for 1 h followed by filtration and centrifugation at 10000 r/min for 15 min. The supernatant was further filter with 0.22 μm nylon membrane for analysis. 20 μL samples and amino acids standard solution (2 nmol) were injected to Hitachi HPLC Packed column (Ion-exchange resin, 4.6 mm i.d., 60 mm length, 3 μm particle size, Tokyo, Japan) with sulfone (SO_3^-) groups as the active exchange site (amino acids were derivatised through ninhydrin reaction within the column) and detected with a UV-visible detector (Hitach High-Technologies Corp.) at a specific wavelength of 570 nm and 440 nm for proline and hydro proline. The

amino acids content in each sample was calculated by comparing each of the peak area with the standard. The analysis was performed in triplicate.

1.3. Sugar analysis

The sugars compositions of the samples were determined using High Performance Ion Chromatography (HPIC) method adopted from Shi et al. (2019) with slightly modifications. Briefly, 1 g of sample was dissolved in 5 mL of ultrapure water, and a further 200 times dilution was applied, and then filtered through 0.45 μm membrane prior to analysis. Dionex 5000 HPLC system equipped with an ED-40 detector were used for the analysis, and CarboPac PA1 column (250 mm × 4.0 mm i.d., 10-μm particle size, Dionex Co., Osaka, Japan) connected to a CarboPac PA1 (50 × 4.0 mm i.d.,) guard column was used as the separation unit. The Dionex GP-40 pump was operated in isocratic mode with 200 mM NaOH at flow rate of 0.6 mL/min and injection volume of 10 μL, the oven was programmed at a constant temperature of 30 °C, the concentration of sugars was calibrated by plotting peak area against concentration for the respective sugar standards. All assays were performed in triplicate.

2. Results and discussion

2.1. Correlation between volatile compounds and precursors

2.1.1. Correlation with FAA _ partial least squares regression (PLSR) analysis

Partial least squares regression (PLSR) analysis was applied to discriminate among the contributing amino acids and to determine the correlation between each volatile species and each of the FAA (Figure S2a). The X variables are the 12 volatile species identified within the process, and the Y variables are the 18 free amino acid. The plot contains two ellipses, of which the outer ellipse is the unit-circle and indicates 100% explained variance, and the inner ellipse indicates 50% explained variance. As shown in Figure S2a, seven volatile compounds and all the amino acids were located in between 50% and 100% explained variance, indicating their variances could be well explained by this PLSR Model except alcohols, ketones, phenols, terpene compounds and others located outside the ellipse.

Variables close to each other in the same quadrant of the loadings plot have a high positive correlation. Aldehydes (1), sulfur compounds (3), acids (8), nitrogen heterocyclic compounds (9), oxygen heterocyclic compounds (10) were characterized with positive factor 1 loadings. However, esters (2), hydrocarbons (7) in diagonally opposed quadrants have a tendency to be negative factor 1 loading. All the identified FAA were positively correlated to 1, 3, 8, 9 and10, while negatively correlated to 2 and 7, indicating the formation of aldehydes, sulfur compounds, acids, oxygen and nitrogen heterocyclic compounds could be tightly correlated to the amino acids, while esters, hydrocarbons formed in the system were not significantly affected by the amino acids.

2.1.2. Correlation with sugars _ partial least squares regression (PLSR) analysis

To further identify the correlation between sugars and the volatile compounds, partial least squares regression (PLSR) analysis was applied to determine the relationships of each volatile species with the sugars (Figure S2b). The X variables are the 12 volatile compounds identified within the process, and the Y variables are the 3 sugars *i.e.*, glucose, fructose, and sucrose. Similar to the amino acids plot, the outer ellipse is the unit-circle and indicates 100% explained variance and the inner ellipse indicates 50% explained variance. The cross-validation interpretation variance is 95%. As shown in Figure S2b, nine volatile compounds (1, 2, 3, 4, 7, 8, 9, 10, 11) and all the sugars were plotted in between the two ellipses, indicating that all their variances could be well explained by this PLSR Model except ketones (5), phenols (6) and others (12) that located outside the ellipse.

For volatile compounds, aldehydes (1), sulfur compounds (3), acids (8), nitrogen heterocyclic compounds (9), and oxygen heterocyclic compounds (10) were characterized with positive factor 1 loadings, however, esters (2), alcohols (4), hydrocarbons (7), and terpene compounds (11) were in diagonally opposed quadrants and had a tendency to be negative factor 1 loadings. There were significant correlations among 1, 9, 10 in the upper-right quadrant and so as 3 and 8 in the lower-right quadrant. Among the 3 sugars, sucrose was positively correlated to 2, 4, 7 and 11, and negatively correlated to 1, 3, 8, 9, 10, while glucose and fructose had a opposite correlations with the volatiles,

indicating volatiles including aldehydes, sulfur compounds, acids, oxygen and nitrogen heterocyclic compounds were positively associated with glucose and fructose and negatively associated with sucrose, further evidence the MRPs were the dominate aroma-active compounds throughout the whole heating and dehydration process.

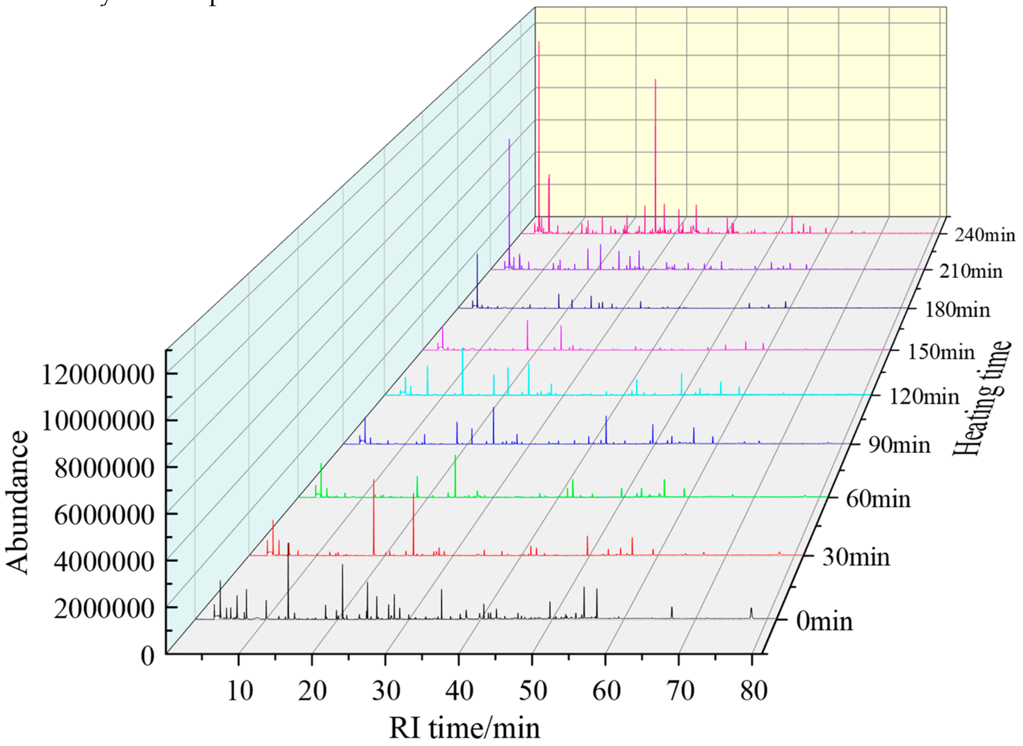

Figure S1. 3D waterfall total ion chromatograms of volatile compounds.

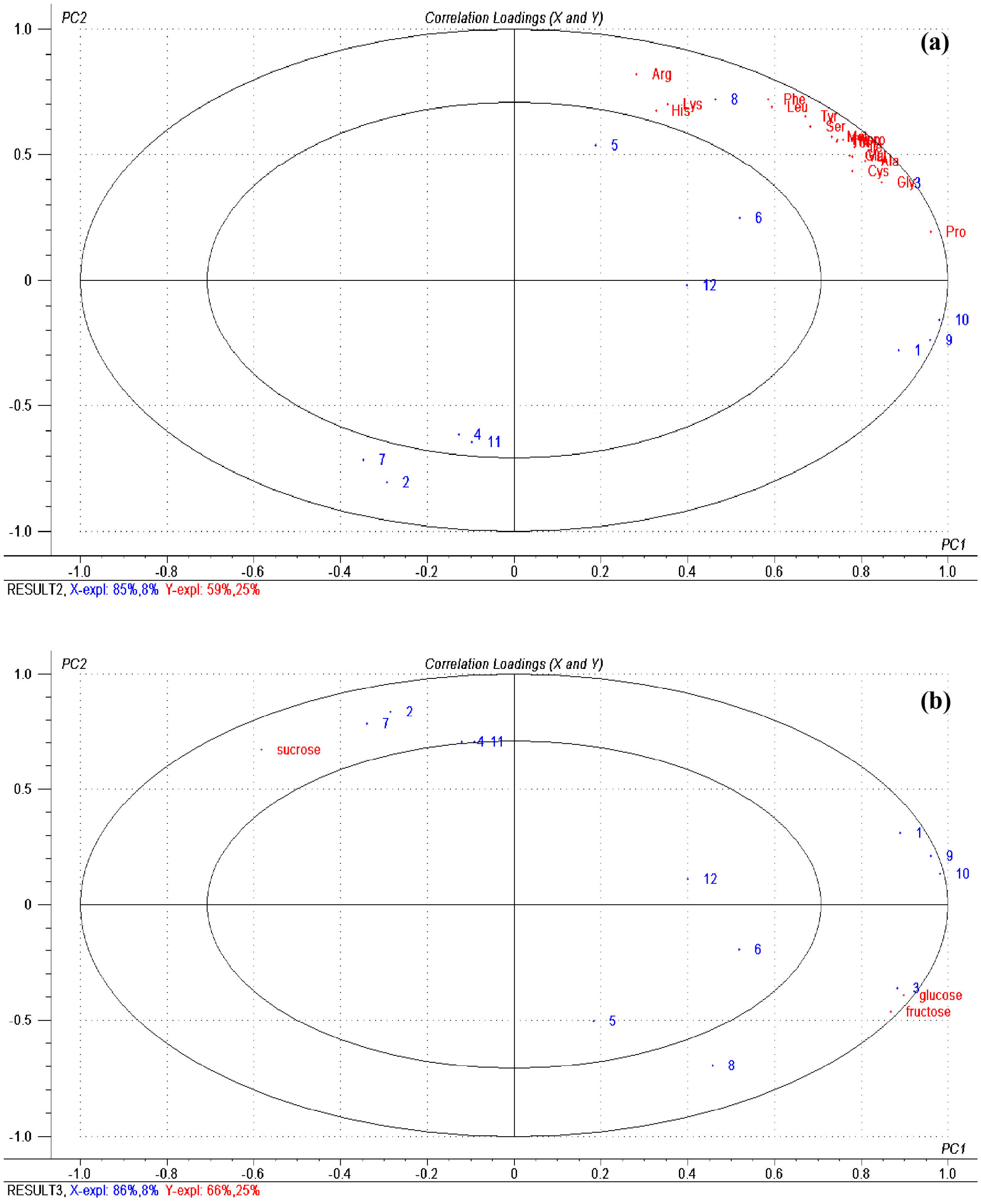

Figure S2. Correlation loadings plot of volatile compounds and (a) free amino acid, and (b) sugars during NCS formation process. (Aldehydes-1, esters-2, sulphur compounds-3, alcohols-4, ketones-5, phenols-6, hydrocarbons-7, acids-8, nitrogen compounds-9, oxygen heterocyclic compounds-10, terpene compounds-11, others-12).

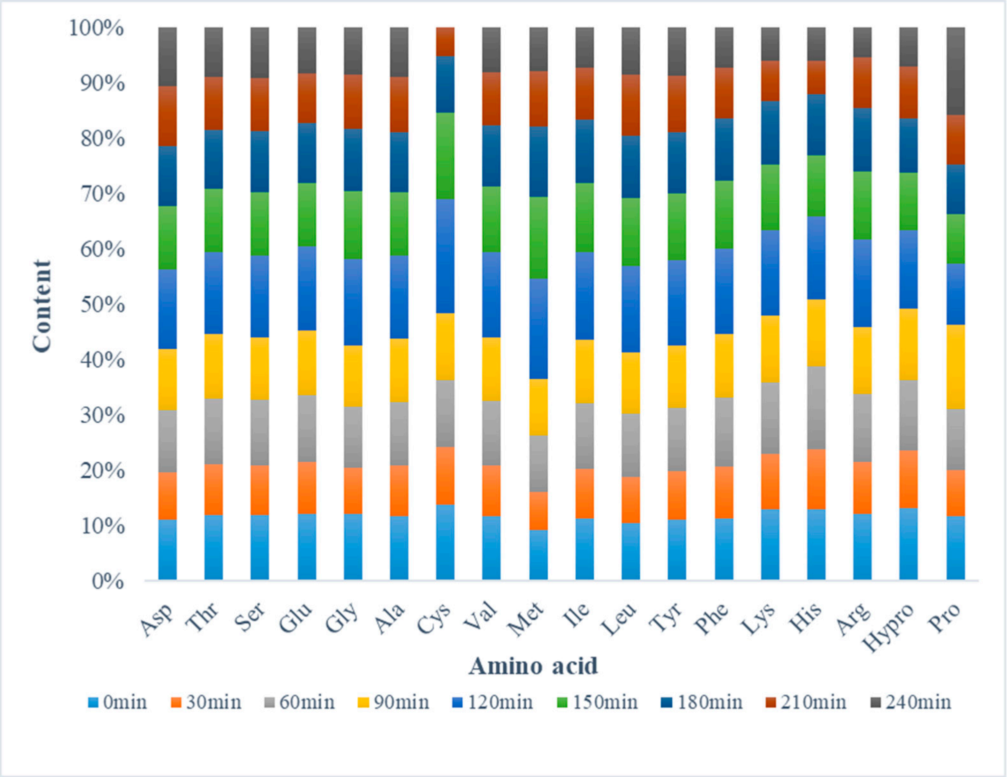

Figure S3. Free amino acids percentage variations at different stages during the dehydration of sugarcane juice to NCS products.

.

Table S1. Volatile compounds and the relative concentrations (μg/g) identified in the interval samples during the NCS formation process using headspace-solid phase microextraction and gas chromatography–mass spectrometry (HS-SPME and GC-MS).

NO.	Compounds	CAS	RI ^{A}	Concentration(μg/g) ^{B} Heating time（min）									Odor thresholds (μg/g)	Odor descriptions	Identification ^{C}
				0	30	60	90	120	150	180	210	240			
**Aldehydes**															
1	2-Methyl-butanal	96-17-3	910	0.30±0.01^{d}	ND	ND	ND	ND	0.41±0.02^{d}	0.85±0.02^{c}	4.9±0.02^{b}	12.07±0.14^{a}	0.0008	Chocolate	RI, MS
2	3-Methyl-butanal	590-86-3	913	0.31±0.01^{c}	0.08±0.00^{c}	0.11±0.00^{c}	0.10±0.01^{c}	0.14±0.03^{c}	0.32±0.00^{c}	0.61±0.09^{c}	4.58±0.90^{b}	14.42±0.85^{a}	0.00025	Bready odor	RI, MS
3	Pentanal	110-62-3	974	2.03±0.05	ND	ND	ND	ND	ND	ND	ND	ND	0.008	Special aroma	RI, MS
4	Hexanal	66-25-1	1087	8.04±0.09^{a}	0.40±0.00^{c}	ND	ND	ND	ND	ND	0.66±0.02^{b}	0.61±0.02^{b}	0.21	Grassy	RI, MS
5	Heptanal	111-71-7	1199	1.63±0.11^{a}	0.19±0.01^{e}	0.20±0.01^{e}	ND	0.30±0.02^{d}	ND	ND	0.58±0.03^{c}	1.29±0.02^{b}	0.01	Fruity	RI, MS
6	(E)-2-hexenal	6728-26-3	1233	0.14±0.01^{a}	0.10±0.02^{b}	ND	ND	ND	ND	ND	ND	ND	0.04	Leaf aroma	RI, MS
7	Octanal	124-13-0	1295	0.75±0.03^{c}	ND	0.26±0.02^{e}	ND	0.35±0.02^{d}	ND	ND	0.84±0.05^{b}	1.29±0.07^{a}	0.0001	Orange, honey	RI, MS
8	Nonanal	124-19-6	1394	1.98±0.04^{c}	0.97±0.16^{f}	1.42±0.06^{d}	1.23±0.04^{e}	1.26±0.09^{de}	1.42±0.05^{d}	1.39±0.15^{de}	3.81±0.09^{a}	3.26±0.05^{b}	0.0035	Sweet orange, peanut flavor	RI, MS
9	(E, E)-2,4-Heptadienal	4313-03-5	1495	1.58±0.01^{a}	0.26±0.00^{b}	ND	ND	ND	ND	ND	ND	ND	-	-	RI, MS
10	Benzaldehyde	100-52-7	1523	3.50±0.07^{a}	1.18±0.02^{c}	ND	ND	ND	0.50±0.01^{d}	ND	ND	1.41±0.02^{b}	0.3	Almond flavor	RI, MS
11	2,6,6-Trimethyl-1-cyclohexene-1-carboxaldehyde	432-25-7	1615	0.30±0.02	ND	ND	ND	ND	ND	ND	ND	ND	-	Cool, fruity and delicate flavor	RI, MS
12	(E, E)-2,4-Nonadienal	5910-87-2	1696	1.18±0.63^{a}	0.16±0.02^{b}	ND	ND	ND	ND	ND	ND	ND	0.00006	-	RI, MS
13	(E, E)-2,4-Decadienal	25152-84-5	1804	1.28±0.07	ND	ND	ND	ND	ND	ND	ND	ND	0.00005	Chicken oil flavor	RI, MS
14	Decanal	112-31-2	1497	ND	1.00±0.02^{f}	1.72±0.08^{c}	1.29±0.06^{d}	1.16±0.03^{e}	1.20±0.07^{de}	0.89±0.04^{g}	1.87±0.02^{b}	2.42±0.10^{a}	0.005	Sweetness, floral	RI, MS
15	Benzene acetaldehyde	122-78-1	1640	ND	0.66±0.01^{c}	ND	ND	ND	ND	ND	5.47±0.11^{a}	3.47±0.14^{b}	0.009	Sweet odor	RI, MS

16	2-Methyl-propanal	78-84-2	816	ND	ND	ND	ND	ND	ND	ND	ND	3.55±0.09	0.0007	Fresh aldehydic odor	RI, MS
17	Methional	3268-49-3	1457	ND	ND	ND	ND	ND	ND	ND	ND	0.38±0.02	0.00004	Bready odor	RI, MS
18	Alpha-ethylidene-benzene acetaldehyde	4411-89-6	1923	ND	ND	ND	ND	ND	ND	ND	ND	0.62±0.02	-	Powdery and cocoa odor	RI, MS
19	3-Ethyl-benzaldehyde	34246-54-3	1701	ND	ND	ND	ND	ND	ND	ND	ND	0.51±0.00	-	Bitter almond odor	RI, MS
**Esters**															
20	Ethyl acetate	141-78-6	886	2.67±0.08^{a}	ND	ND	ND	ND	ND	ND	ND	0.36±0.01^{b}	0.1	Etherial, fruity and rum-like odor	RI, MS
21	Formic acid, octyl ester	112-32-3	1554	1.31±0.01	ND	ND	ND	ND	ND	ND	ND	ND	-	Fruity odor	RI, MS
22	2,2,4-Trimethyl-1,3-pentanedioldiisobutyrate	6846-50-0	1869	5.60±0.09^{b}	4.45±0.16^{d}	4.08±0.08^{e}	7.21±0.05^{a}	4.70±0.02^{c}	ND	ND	ND	1.00±0.09^{f}	-	-	RI, MS
23	Octadecanoic acid, methyl ester	112-39-0	2249	0.24±0.01^{b}	ND	ND	0.60±0.02^{a}	ND	ND	ND	ND	ND	-	Waxy, fruity	RI, MS
24	Octadecanoic acid, ethyl ester	628-97-7	2251	0.68±0.05^{b}	ND	ND	1.16±0.05^{a}	ND	ND	ND	ND	0.29±0.02^{c}	-	Creamy odor	RI, MS
25	Dibutyl phthalate	84-74-2	2680	5.14±0.03	ND	ND	ND	ND	ND	ND	ND	ND	-	Faint odor	RI, MS
26	2-Ethylhexyl ester benzoic acid	5444-75-7	2161	ND	ND	1.35±0.10^{b}	ND	1.80±0.02^{a}	ND	ND	ND	ND	-	-	RI, MS
27	Phthalic acid, isobutyl octyl ester	1000309-04-5	-	ND	ND	ND	2.20±0.14	ND	ND	ND	ND	ND	-	-	MS
28	Methyl salicylate	119-36-8	1769	ND	ND	ND	ND	ND	ND	ND	ND	0.23±0.02	0.06	Minty odor	RI, MS
29	2-Furanmethanolacetate	623-17-6	1535	ND	ND	ND	ND	ND	ND	ND	ND	1.15±0.03	-	Banana-like odor	RI, MS
**Sulfur compounds**															
30	Dimethyl sulfide	75-18-3	753	5.11±0.20^{e}	5.38±0.22^{e}	4.98±0.11^{e}	3.91±0.14^{f}	3.16±0.17^{g}	6.70±0.12^{d}	25.40±0.20^{c}	46.15±0.71^{b}	50.49±0.54^{a}	0.002	Stink	MS
31	Dimethyl Sulfoxide	67-68-5	1578	0.13±0.03^{d}	0.11±0.01^{d}	0.07±0.01^{d}	0.07±0.01^{d}	0.06±0.01^{d}	0.08±0.01^{d}	0.72±0.09^{b}	0.58±0.02^{c}	0.94±0.05^{a}	-	Bitterness	RI, MS
**Alcohols**															
32	Ethanol	64-17-5	928	5.23±0.12^{a}	2.58±0.07^{b}	ND	ND	ND	ND	ND	1.74±0.02^{c}	ND	2900	Wine, pungent, spicy taste	RI, MS

33	2-Heptanol	543-49-7	1325	2.43±0.24^{a}	0.25±0.00^{b}	ND	ND	ND	ND	ND	ND	ND	0.1	Unpleasant special smell	RI, MS
34	1-Hexanol	111-27-3	1356	1.49±0.04	ND	ND	ND	ND	ND	ND	ND	ND	0.2	Waxy odor	RI, MS
35	1-Octen-3-ol	3391-86-4	1450	5.79±0.10^{a}	0.32±0.02^{b}	ND	ND	ND	ND	ND	ND	ND	0.002	Earthy odor	RI, MS
36	2-Ethyl-1-hexanol	104-76-7	1487	3.97±0.12^{a}	0.45±0.03^{e}	0.33±0.03^{f}	0.65±0.01^{d}	0.47±0.07^{e}	0.35±0.01^{f}	ND	1.10±0.08^{c}	1.79±0.05^{b}	-	Green scent	RI, MS
37	2-Nonanol	628-99-9	1516	0.30±0.01	ND	ND	ND	ND	ND	ND	ND	ND	0.28	Waxy, green, creamy, orange, citrus, fruity	RI, MS
38	Cis-1-methyl-4-(1-methylethyl)-2-cyclohexen-1-ol	29803-82-5	1598	0.30±0.01	ND	ND	ND	ND	ND	ND	ND	ND	-	-	RI, MS
39	4-Isopropyl-1-methylcyclohex-2-enol	29803-82-5	1560	0.30±0.00	ND	ND	ND	ND	ND	ND	ND	ND	-	-	RI, MS
40	Menthol	89-78-1	1634	0.20±0.00	ND	ND	ND	ND	ND	ND	ND	ND	1	Mint aroma	RI, MS
41	1-Nonanol	143-08-8	1652	0.63±0.02	ND	ND	ND	ND	ND	ND	ND	ND	0.002	-	RI, MS
42	Benzyl alcohol	100-51-6	1867	ND	ND	ND	ND	ND	ND	0.23±0.04	ND	ND	5.5	Aromatic	RI, MS
43	1-Octanol	111-87-5	1553	ND	0.11±0.02^{c}	ND	ND	ND	ND	ND	0.38±0.05^{b}	0.43±0.05^{a}	0.054	Rose fragrance	RI, MS
44	Cedrol	77-53-2	2105	0.67±0.03^{a}	ND	0.34±0.02^{c}	0.67±0.04^{a}	0.41±0.02^{b}	ND	ND	ND	ND	-	Spicy	RI, MS
**Ketones**															
45	2-Heptanone	110-43-0	1198	0.49±0.00^{a}	ND	ND	ND	ND	ND	ND	0.24±0.01^{b}	0.16±0.00^{c}	0.68	Pear-like aroma	RI, MS
46	3-Octanone	106-68-3	1263	0.33±0.03	ND	ND	ND	ND	ND	ND	ND	ND	1	Lavender-type fragrance	RI, MS
47	2-Octanone	111-13-7	1292	4.07±0.12^{f}	4.35±0.14^{e}	4.51±0.25^{e}	5.30±0.27^{d}	5.44±0.14^{d}	7.39±0.16^{c}	10.80±0.10^{b}	11.70±0.08^{a}	1.47±0.04^{g}	0.05	Immature apple scent	RI, MS
48	2-Nonanone	821-55-6	1391	0.30±0.01	ND	ND	ND	ND	ND	ND	ND	ND	0.05	Fruity, sweet, waxy, soapy, green, coconut, and creamy	RI, MS
49	3-Nonen-2-one	14309-57-0	1511	0.30±0.02^{c}	ND	0.10±0.01^{d}	ND	ND	ND	ND	0.71±0.10^{b}	1.44±0.11^{a}	-	-	RI, MS
50	(E)-1-(2,6,6-trimethyl-1,3-cyclohexadien-1-yl)-2-buten-1-one	23726-93-4	1812	0.25±0.01^{e}	0.26±0.02^{e}	0.32±0.0^{de}	0.36±0.00^{d}	ND	0.70±0.01^{c}	ND	3.23±0.03^{b}	4.67±0.13^{a}	0.00000495	-	RI, MS

51	(E)-6,10-dimethyl-5,9-undecadien-2-one	3796-70-1	1846	1.72±0.06^{c}	1.70±0.10^{c}	1.88±0.05^{b}	1.69±0.08^{c}	1.20±0.10^{d}	0.94±0.06^{e}	1.01±0.10^{e}	3.04±0.08^{a}	ND	0.01	Floral	RI, MS
52	Beta-ionone	79-77-6	1930	0.27±0.01	ND	ND	ND	ND	ND	ND	ND	ND	0.00567	Cypress, raspberry aroma	RI, MS
53	2,6-Bis(1,1-dimethylethyl)-4-hydroxy-4-methyl-2,5-cyclohexadien-1-one	1000401-12-0	2088	2.16±0.05^{d}	2.56±0.21^{c}	1.94±0.09^{e}	4.53±0.10^{b}	10.66±0.17^{a}	ND	ND	ND	0.60±0.04^{f}	-	-	RI, MS
54	6-Methyl-5-hepten-2one	110-93-0	1343	ND	0.27±0.01^{d}	0.43±0.03^{b}	ND	ND	0.33±0.01^{c}	ND	1.25±0.07^{a}	ND	-	Fruity, fresh fragrance	RI, MS
55	2-Pentadecanone	2345-28-0	2009	ND	ND	ND	0.34±0.01	ND	ND	ND	ND	ND	-	-	RI, MS
56	2-Nonen-4-one	32064-72-5	1480	ND	ND	ND	ND	ND	ND	ND	0.48±0.02^{b}	1.21±0.02^{a}	-	-	RI, MS
57	Cyclopent-4-ene-1,3-dione	1000411-44-6	1585	ND	ND	ND	ND	ND	ND	ND	0.45±0.02^{b}	1.57±0.07^{a}	-	-	RI, MS
58	4-Hydroxy-3methyl acetophenone	876-02-8	1996	ND	ND	ND	ND	ND	ND	ND	ND	0.27±0.02	-	-	RI, MS
**Phenols**															
59	Butylated hydroxytoluene	128-37-0	1901	0.95±0.05^{c}	0.76±0.01^{d}	0.63±0.02^{e}	0.97±0.01^{c}	1.87±0.10^{a}	0.63±0.01^{e}	1.31±0.05^{b}	1.32±0.01^{b}	ND	-	-	RI, MS
60	3-Allyl-6-methoxyphenol	501-19-9	2154	0.10±0.00	ND	ND	ND	ND	ND	ND	ND	ND	-	-	RI, MS
61	2,4-Di-tert-butylphenol	96-76-4	2307	6.70±0.06^{a}	2.76±0.10^{d}	3.65±0.20^{c}	4.40±0.06^{b}	4.32±0.32^{b}	3.30±0.21^{c}	2.80±0.30^{d}	4.43±0.24^{b}	2.65±0.23^{d}	-	-	RI, MS
62	2-Methoxy-4-vinylphenol	7786-61-0	2184	ND	1.05±0.13^{e}	2.00±0.05^{d}	2.29±0.13^{c}	1.88±0.04^{d}	1.96±0.03^{d}	3.81±0.13^{b}	3.81±0.20^{b}	5.92±0.08^{a}	0.01	Woody odor	RI, MS
63	Eugenol	97-53-0	2155	ND	1.07±0.06	ND	ND	ND	ND	ND	ND	ND	0.001	Spicy odor	RI, MS
**Hydrocarbons**															
64	Beta-Phellandrene	555-10-2	1214	0.32±0.02	ND	ND	ND	ND	ND	ND	ND	ND	0.036	Minty scent	RI, MS
65	3-Ethyl-2-methyl-1,3-hexadiene	61142-36-7	1417	2.29±0.16^{a}	0.30±0.01^{b}	0.11±0.02^{c}	ND	ND	ND	ND	ND	ND	-	-	RI, MS
66	Methylene chloride	75-09-2	922	0.34±0.43^{a}	0.07±0.02^{ab}	0.11±0.01^{ab}	0.10±0.01^{ab}	0.15±0.01^{ab}	0.11±0.00^{ab}	0.11±0.02^{ab}	0.12±0.02^{ab}	ND	24	Pungent smell	RI, MS
67	Pentadecane	629-62-9	1491	0.21±0.01^{b}	0.09±0.01^{d}	0.26±0.01^{a}	0.20±0.01^{b}	0.12±0.02^{c}	ND	ND	0.25±0.03^{a}	ND		-	RI, MS
68	Hexadecane	544-76-3	1589	0.36±0.01^{a}	0.15±0.00^{d}	0.30±0.01^{c}	0.11±0.00^{e}	0.30±0.01^{b}	ND	ND	ND	ND	-	-	RI, MS

69	Heptadecane	629-78-7	1686	0.42±0.01[a]	ND	0.13±0.01[b]	ND	ND	ND	ND	ND	ND	-	-	RI, MS
70	Nonadecane	629-92-5	1884	0.68±0.02[a]	ND	ND	0.37±0.02[b]	ND	ND	ND	ND	ND	-	-	RI, MS
71	Cyclododecane	294-62-2	1955	1.47±0.02[a]	0.99±0.01[cd]	1.20±0.05[ab]	0.86±0.63[cd]	0.63±0.03[c]	ND	ND	ND	ND	-	-	RI, MS
72	Eicosane	112-95-8	1983	0.61±0.03	ND	ND	ND	ND	ND	ND	ND	ND	-	-	RI, MS
73	Nonyl-cyclopropane	74663-85-7	1954	ND	ND	ND	ND	ND	ND	ND	ND	0.03±0.02	-	-	RI, MS
**Acids**													-		
74	2-Propenoic acid	79-10-7	1630	ND	ND	ND	0.14±0.01[f]	0.36±0.04[e]	1.58±0.01[d]	6.06±0.06[c]	4.15±0.09[a]	2.54±0.05[b]	-	Pungent smell	RI, MS
75	Acetic acid	64-19-7	1453	ND	ND	0.17±0.02[g]	0.64±0.03[f]	0.90±0.09[e]	1.81±0.08[d]	4.63±0.05[a]	2.60±0.17[b]	2.00±0.04[c]	120	Acidic odor	RI, MS
76	3-Hydroxy-2,2,4-trimethylpentylester 2-methyl-propanoic acid	77-68-9	1860	ND	ND	0.12±0.01	ND	ND	ND	ND	ND	ND	-	-	RI, MS
77	Nonanoic acid	112-05-0	2150	ND	ND	ND	0.32±0.01	ND	ND	ND	ND	ND	1.5	Special smell	RI, MS
78	3-Chloro-2-methoxypyridine-5-boronic acid	942-43-8	1474	ND	ND	ND	ND	ND	ND	ND	ND	0.97±0.08	-	-	RI, MS
**Nitrogen heterocyclic compounds**															
79	3-Methyl-2,5-piperazinedione	6062-46-0	2022	ND	ND	ND	ND	ND	ND	ND	ND	0.52±0.00	-	-	RI, MS
80	1-(1H-pyrrol-2-yl)-ethanone	932-16-1	1962	ND	ND	ND	ND	ND	ND	ND	0.54±0.05[b]	1.44±0.09[a]	-	Coffee, fruit aroma	RI, MS
81	2,6-Dimethyl-pyrazine	108-50-9	1334	ND	ND	ND	ND	ND	ND	ND	0.58±0.02[b]	3.55±0.08[a]	10	Roasted nutty odor	RI, MS
82	Methyl-pyrazine	109-08-0	1276	ND	ND	ND	ND	ND	ND	ND	ND	3.00±0.13	1	Nutty chocolate odor	RI, MS
83	2,3-Dimethyl-pyrazine	5910-89-4	1351	ND	ND	ND	ND	ND	ND	ND	MD	0.66±0.03	0.1	Nutty and peanut butter odor	RI, MS
84	2-Ethyl-5-methyl-pyrazine	13360-64-0	1393	ND	ND	ND	ND	ND	ND	ND	ND	0.46±0.03	1	Nutty and roasted coffee odor	RI, MS
85	3-Ethyl-2,5-dimethyl-pyrazine	13360-65-1	1445	ND	ND	ND	ND	ND	ND	ND	ND	0.53±0.02	0.05	Nutty roasted odor	RI, MS

86	Ethyl-pyrazine	13925-00-3	1339	ND	ND	ND	ND	ND	ND	ND	ND	1.07±0.07	430	Nutty odor	RI, MS
87	2-Ethyl-6-methyl-pyrazine	13925-03-6	1387	ND	ND	ND	ND	ND	ND	ND	ND	1.05±0.07	860	Nutty odor	RI, MS
**Oxygen heterocyclic compounds**															
88	Dihydro-2-methyl-3(2H)-furanone	3188-00-9	1274	ND	ND	ND	ND	ND	ND	ND	ND	0.10±0.01	-	Caramel odor	RI, MS
89	2,3-Dihydro-3,5-dihydroxy-6-methyl-4H-pyran-4-one	28564-83-2	2130	ND	ND	ND	ND	ND	ND	ND	1.81±0.06b	3.23±0.04a	200	Sweet caramel	RI, MS
90	1-(2-Furanyl)-ethanone	1192-62-7	1505	ND	ND	ND	ND	ND	ND	ND	ND	9.67±1.07	-	Coffee, potato chips	RI, MS
91	Furfural	98-01-1	1467	ND	0.25±0.01^{gh}	0.66±0.01^{fg}	0.92±0.04^{ef}	1.23±0.07^{e}	2.25±0.32^{d}	5.04±0.23^{c}	8.76±0.23^{b}	56.14±0.65^{a}	8	Chocolate odor	RI, MS
92	2-Furanmethanol	98-00-0	1655	ND	ND	ND	ND	ND	ND	0.54±0.07^{c}	1.47±0.10^{b}	9.92±0.13^{a}	1	Bready odor	RI, MS
93	5-Methyl-2-furan methanol	3857-25-8	1714	ND	ND	ND	ND	ND	ND	ND	0.83±0.06^{b}	2.10±0.16^{a}	-	Bready odor	RI, MS
94	5-methyl-2-Furancarboxaldehyde	620-02-0	1573	ND	ND	ND		ND	ND	ND	1.02±0.13^{b}	9.46±0.09^{a}	0.05	Caramel odor	RI, MS
95	5-Hydroxymethylfurfural	67-47-0	2490	ND	ND	ND	ND	ND	ND	ND	ND	1.52±0.09	630	Buttery and caramel odor	RI, MS
96	2,3-dihydro-benzofuran	496-16-2		ND	1.64±0.13^{d}	ND	ND	3.36±0.03^{a}	ND	ND	3.25±0.06^{b}	2.46±0.07^{c}		-	MS
97	2-Pentyl-furan	3777-69-3	1240	0.29±0.01^{e}	0.12±0.02^{f}	0.24±0.02^{ef}	0.25±0.03^{ef}	0.29±0.01^{e}	0.51±0.11^{d}	0.76±0.13^{c}	2.53±0.07^{b}	4.12±0.10^{a}	0.0048	Earthy, fruity odor	RI, MS
98	2-Ethyl-furan	3208-16-0	944	ND	ND	ND	ND	ND	0.14±0.01^{c}	0.25±0.01^{b}	0.32±0.04^{a}	0.32±0.01^{a}	-	Flue-cured tobacco	RI, MS
99	2-Methyl-furan	534-22-5	869	ND	ND	ND	ND	ND	ND	ND	0.30±0.02^{a}	0.27±0.01^{b}	8200	Chocolate	RI, MS
100	2-Vinylfuran	1487-18-9	1077	ND	ND	ND	ND	ND	ND	ND	ND	0.21±0.01	-	Spicy odor	RI, MS
101	2-n-Butyl furan	4466-24-4	1140	ND	ND	ND	ND	ND	ND	ND	ND	0.54±0.01		Spicy odor	RI, MS
102	2,2'-Bifuran	5905-00-0	1591	ND	ND	ND	ND	ND	ND	ND	ND	3.98±0.06	-	-	RI, MS
103	3-Phenyl-furan	13679-41-9	1844	ND	ND	ND	ND	ND	ND	ND	ND	4.07±0.08	-	Spicy odor	RI, MS
**Terpene compounds**															

104	D-Limonene	5989-27-5	1205	0.43±0.01^{a}	0.22±0.00^{d}	0.25±0.01^{c}	0.29±0.01^{b}	ND	0.19±0.01^{e}	ND	0.21±0.01^{e}	0.20±0.01^{e}	0.034	Sweet orange and citrus, fragrance like flowers	RI, MS
105	1-Methyl-4-(1-methylethyl)-1,3-cyclohexadiene	99-86-5	1189	0.08±0.00	ND	ND	ND	ND	ND	ND	ND	ND	-	Citrus and lemon aroma	RI, MS
**Other compounds**															
106	1,2,3,4-Tetrahydro-1,6,8-trimethyl-naphthalene	30316-36-0	1475	ND	ND	ND	ND	ND	ND	ND	ND	0.88±0.02	-	-	RI, MS
107	2-(2-Butenyl)-1,3,5-trimethyl-benzene	63435-25-6	1514	ND	ND	ND	ND	ND	ND	ND	ND	0.28±0.01	-	-	RI, MS
108	1,2,3,4-Tetrahydro-1,1,6-Trimethyl-naphthalene	475-03-6	1660	ND	ND	ND	ND	ND	ND	ND	0.40±0.03^{b}	0.80±0.04^{a}	-	-	RI, MS
109	2,2',5,5'-Tetramethyl-1,1'-biphenyl	3075-84-1		ND	ND	0.49±0.06^{b}	0.25±0.06^{c}	ND	ND	ND	0.71±0.02^{a}	ND	-	-	MS
110	(E)-1,2,3-Trimethyl-4-propenyl-naphthalene	26137-53-1	2177	4.16±0.05^{a}	ND	0.45±0.03^{d}	0.83±0.02^{b}	0.38±0.01^{de}	ND	0.36±0.05^{c}	ND	0.36±0.05^{e}	-	-	RI, MS
111	Methoxy-phenyl-oxime	1000222-86-6	1754	ND	0.83±0.01^{e}	1.38±0.09^{d}	0.64±0.06^{f}	ND	1.84±0.05^{c}	2.47±0.10^{a}	2.28±0.11^{b}	0.88±0.05^{e}	-	-	RI, MS

Each value is the mean of triplicate determinations ± standard deviation and values with different letter within the same row are significantly different ($p<0.05$). ND not detected. ^{A} Retention indices relative to n-alkanes (C8-C40) on a polar DB-Wax column; ^{B} Integration of the peak was performed by choosing the characteristic ion (m/z) for the respective compounds with the highest matching index (>80%); as to avoid possible interference by other compounds. The concentrations are given in terms of equivalents to the internal standard 2-octanol; ^{C} Identification based on retention index (RI), NIST 14 MS library (MS).

References

1. Huang, S., 2019, A preliminary study on the identification of aroma components of brown sugar and the tracebility, master thesis, Guangxi University, Nanning.
2. Shi, C.; Rackemann, D.W.; Moghaddam, L.; Wei, B.; Li, K.; Lu, H.; Xie, C.; Hang, F.; Doherty, W.O.S. Ceramic membrane filtration of factory sugarcane juice: Effect of pretreatment on permeate flux, juice quality and fouling. *J Food Eng.*, **2019**, *243*, 101-113. https://doi.org/10.1016/j.jfoodeng.2018.09.012.
3. Shim, Y.S.; Yoon, W.J.; Ha, J.; Seo, D.; Lee, K.W.; Lee, W.Y.; Kwon, K.; Kang, T.S.; Lee, J.H.; Kim, H.J.; et al. Method validation of 16 types of structural amino acids using an automated amino acid analyzer. *Food Sci. Biotechnol.*, **2013**, *22(6)*, 1567–1571. https://doi.org/10.1007/s10068-013-0252-0.
